# Separation of Branched Alkanes Feeds by a Synergistic Action of Zeolite and Metal‐Organic Framework

**DOI:** 10.1002/advs.202201494

**Published:** 2022-06-05

**Authors:** Pedro F. Brântuas, Adriano Henrique, Mohammad Wahiduzzaman, Alexander von Wedelstedt, Tanmoy Maity, Alírio E. Rodrigues, Farid Nouar, U‐Hwang Lee, Kyung‐Ho Cho, Guillaume Maurin, José A. C. Silva, Christian Serre

**Affiliations:** ^1^ Centro de Investigação de Montanha (CIMO) Instituto Politécnico de Bragança Campus de Santa Apolónia Bragança 5300‐253 Portugal; ^2^ LSRE‐LCM ‐ Laboratory of Separation and Reaction Engineering – Laboratory of Catalysis and Materials Faculty of Engineering University of Porto Rua Dr. Roberto Frias Porto 4200‐465 Portugal; ^3^ ICGM University of Montpellier CNRS, ENSCM, Montpellier Montpellier 34095 France; ^4^ Tata Institute of Fundamental Research‐Hyderabad Hyderabad Telangana 500046 India; ^5^ Institut des Matériaux Poreux de Paris ESPCI Paris Ecole Normale Supérieure de Paris CNRS PSL University Paris 75005 France; ^6^ Research Group for Nanocatalysts (RGN) and Chemical and Process Technology Division Korea Research Institute of Chemical Technology (KRICT) Gajeong‐Ro 141 Yuseong Daejeon 34114 Republic of Korea

**Keywords:** gasoline, metal‐organic frameworks (MOFs), separation, simulation, zeolite

## Abstract

Zeolites and metal‐organic frameworks (MOFs) are considered as “competitors” for new separation processes. The production of high‐quality gasoline is currently achieved through the total isomerization process that separates pentane and hexane isomers while not reaching the ultimate goal of a research octane number (RON) higher than 92. This work demonstrates how a synergistic action of the zeolite 5A and the MIL‐160(Al) MOF leads to a novel adsorptive process for octane upgrading of gasoline through an efficient separation of isomers. This innovative mixed‐bed adsorbent strategy encompasses a thermodynamically driven separation of hexane isomers according to the degree of branching by MIL‐160(Al) coupled to a steric rejection of linear isomers by the molecular sieve zeolite 5A. Their adsorptive separation ability is further evaluated under real conditions by sorption breakthrough and continuous cyclic experiments with a mixed bed of shaped adsorbents. Remarkably, at the industrially relevant temperature of 423 K, an ideal sorption hierarchy of low RON over high RON alkanes is achieved, i.e., *n*‐hexane ≫ *n*‐pentane ≫ 2‐methylpentane > 3‐methylpentane ⋙ 2,3‐dimethylbutane > isopentane ≈ 2,2‐dimethylbutane, together with a productivity of 1.14 mol dm^−3^ and a high RON of 92, which is a leap‐forward compared with existing processes.

## Graphical abstract

A synergistic action of the bioderived Al‐dicarboxylate metal‐organic framework MOF MIL‐160(Al) and zeolite 5A enables an efficient thermodynamic/size exclusion separation of pentane and hexane isomers into fractions of low and high research octane number and leads to an exceptional ideal productivity of 1.14 mol dm^−3^ and a high RON of 92.

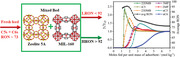

## 1 Introduction

Purifying hydrocarbons is of great importance in petrochemical chemistry and will still remain one of the most challenging and energy‐intensive separation processes in the coming decades until a complete shift toward renewable carbon pathways is found.^[^ ^1^ ^]^ The conventional total isomerization process (TIP) developed by the universal oil products (UOP) for improving the octane rating of light hydrocarbon fractions, especially mixed feedstocks containing pentane and hexane isomers, is among the first and most successful adsorption processes applied in industry.^[^ ^2^ ^]^ The quality of gasoline is evaluated by its “antiknocking” effect in spark‐ignition internal combustion engines measured by the research octane number (RON), i.e., the most common octane rating worldwide shown on the pumps. Gasoline should have at least an RON higher than 92 for its use in modern automotive combustion engines. Typically, the light naphtha, with low RON undergoes an incomplete catalytic isomerization that generates an effluent containing unconverted linear paraffins, mostly *n*‐pentane (*n*‐C5; RON 61.7) and *n*‐hexane (*n*‐C6; RON 30), mixed with their respective branched isomers, i.e., isopentane (i‐C5; RON 93.5), 2‐methylpentane (2MP; RON 74.5), 3‐methylpentane (3MP; RON 75.5), 2,3‐dimethylbutane (23DMB; RON 105) and 2,2‐dimethylbutane (22DMB; RON 94). These remaining linear paraffins are commonly separated from their branched isomers in an adsorption unit packed with zeolite 5A owing to its extraordinary molecular sieving ability of linear from branched paraffins.^[^ ^3^ ^]^ This results in a final isomerate product with an average RON ≈ 87–90.^[^ ^4^ ^]^ However, with the actual process, the monobranched hexane isomers 2MP and 3MP still represent, according to UOP^[^ ^5^ ^]^ and Axens,^[^ ^6^ ^]^ up to 30% of the final product composition. Axens attempted to improve the TIP process by coupling a deisohexanizer column to the adsorption unit for recycling 2MP and 3MP back to the isomerization reactor, to reach a final product (RON > 90) that meets the industrial demand (the Hexorb process).^[^ ^7^ ^]^ However, this technology requires an investment of over US$ 20 million since the distillation column needs a large number of theoretical plates to fractionate the low RON monobranched (2MP, 3MP) from the high RON dibranched (22DMB, 23DMB) hexane isomers and i‐C5 due to their similar boiling points and therefore narrow selectivity.^[^ ^7^ ^]^

One alternative path to make a leap forward in this field would be to find an optimal adsorbent with outstanding alkane isomer separation performance according to classes of high (HRON > 90) and low RON (LRON < 90) compounds. To date, extensive effort has been primarily deployed to assess the separation performance of zeolites with different pore sizes/shapes.^[^ ^8^, ^9^ ^]^ Typically, zeolite BETA was demonstrated to partially discriminate mono and dibranched hexane isomers via a thermodynamically controlled separation mechanism.^[^ ^10^ ^]^ On the other hand, silicalite (MFI) was shown to enable a kinetically driven separation of branched hexanes owing to a slow diffusion of the dibranched isomers in its pore structure.^[^ ^11^ ^]^

More recently, ordered porous hybrid materials, MOFs,^[^ ^12^, ^13^, ^14^, ^15^, ^16^ ^]^ have been proposed as alternative candidates to address this challenging separation owing to the great tunability of their pore size/shape and surface functionality.^[^ ^17^, ^18^ ^]^ Fe_2_(BDP)_3_,^[^ ^19^ ^]^ Zr‐abtc,^[^ ^20^ ^]^ and MIL‐140B^[^ ^21^ ^]^ were revealed to discriminate the hexane isomers into valuable fractions according to the degree of branching through a thermodynamically controlled separation mechanism governed by distinctive host/hexane isomer interactions. Other MOFs including MIL‐53(Fe)‐(CF_3_)_2_,^[^ ^22^ ^]^ Ca(H_2_tcpb),^[^ ^23^ ^]^ and Al‐bttotb^[^ ^24^ ^]^ showed attractive separation performance of dibranched from monobranched hexane isomers via either kinetically driven or molecular sieving‐controlled mechanisms.

However, so far none of the existing zeolites or MOFs have reached optimum performance to meet the industrial needs in terms of separation of complex pentane and hexane feeds, including the benchmark Fe_2_(BDP)_3_ MOF where the HRON i‐C5 elute practically simultaneously with the LRON 2MP and 3MP hexanes.^[^ ^19^ ^]^ Furthermore, MOFs are usually rather expensive, hardly scalable and sometimes air moisture unstable, and in sharp contrast to zeolites^[^ ^5^, ^6^ ^]^ they have not yet been tested under relevant industrial conditions, i.e., with the consideration of complex isomer mixtures and the use of shaped materials as well as under continuous adsorption cyclic operation tests.

**Figure 1:**
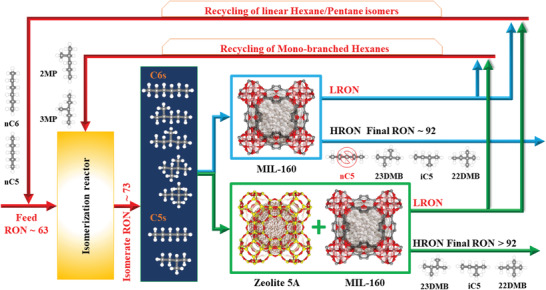
Schematic representation of the recycling technology for TIP processes. Upgraded TIP process using a mixed adsorption bed of shaped MOF‐MIL‐160(Al) and binder‐free zeolite 5A operating under continuous cycling pressure swing adsorption (PSA) to fractionate the hexane isomers reactor output into a final HRON (>92) isomerate product and an LRON recycle effluent fraction back to the reactor.

Therefore, there is still a critical demand to identify an effective single sorbent or a synergy effect of mixed‐bed sorbents with excellent hexane and pentane isomer separation performance for a complete fractionation of the TIP reactor effluent into HRON and LRON fractions under working conditions. In a first step to identify the best sorbent candidate for such an ambitious target, we deliberately selected the Al dicarboxylate MIL‐160(Al) MOF owing to its many attractive features: i) a biosourced linker from the oxidation of 5‐(hydroxymethyl)furan‐2‐carbaldehyde (HMF), that is considered by the bioplastic industry to produce polyethylene furanoate, ii) a green ambient pressure method in line with a facile industrial production,^[^ ^25^ ^]^ iii) its chemical and thermal robustness,^[^ ^26^, ^27^ ^]^ and iv) its microporous channels with a 5.8 Å pore size effective for the separation of small organic molecules.^[^ ^28^ ^]^ Herein, we demonstrated through preliminary breakthrough fixed bed experiments performed on powder and shaped MOF samples that MIL‐160(Al) enables not only a thermodynamically controlled separation of all the C6 isomers but also the discrimination of most of the pentane and hexane alkanes into valuable HRON and LRON fractions according to the degree of branching. However, an undesirable near‐simultaneous elution of *n*‐C5 and 23DMB was observed in the pentane and hexane isomers separation leading to a productivity value for a fixed RON of 92 (around 40%) much lower than that achieved for the separation of hexane isomers only. Decisively, we revealed that this issue can be overcome by a synergistic action of MIL‐160(Al) with the molecular sieve commercially available zeolite 5A in a mixed bed to attain a full separation of all C5/C6 isomers into the desired valuable HRON and LRON fractions. We further delivered a proof of concept with the integration of this highly stable, bioderived, and easily scalable green MOF material in its shaped form with a binder‐free zeolite 5A into a new advanced recycling pressure swing adsorption (PSA) technology for TIP processes (**Figure** **1**) that achieves an unprecedented average RON higher than 92 for a feed containing all of the pentane and hexane isomers. This resulting process with considerable potential for the refining industry relies on the complementary role played by the MOF (thermodynamic‐separation) and the zeolite (molecular sieving) integrated in the mixed bed to effectively separate complex pentane and hexane mixtures into HRON (i‐C5/23DMB/22DMB) and LRON (*n*‐C5/*n*‐C6/2MP/3MP) fractions.

## 2 Results and Discussion

### 2.1 MIL‐160(Al) for Separating All Hexane Isomers into Classes of HRON and LRON Fractions

**Figure 2:**
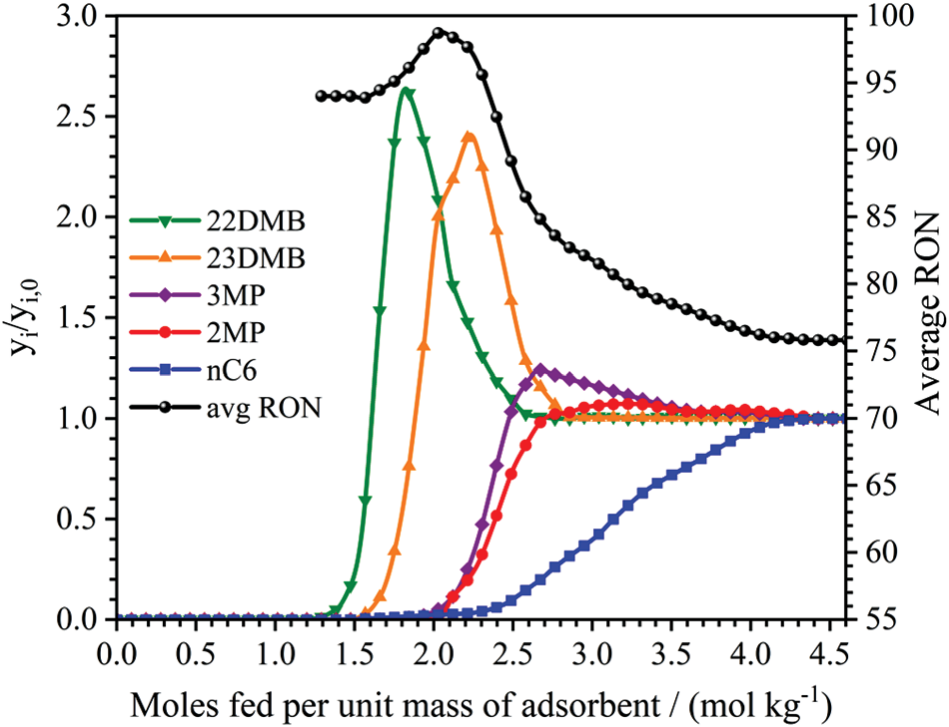
Separation of equimolar mixtures of hexane isomers by fixed bed adsorption with MIL‐160(Al) at 423 K. Breakthrough data plotted as normalized molar fraction of hexane isomers (left *y*‐axis) and average real time RON (right *y*‐axis) as a function of moles fed per unit mass of pure powder MIL‐160(Al) at 423 K and total isomer pressure of 50 kPa.

The core of our proposed technology is based on the outstanding property of MIL‐160(Al) to thermodynamically separate mixtures of hexane isomers according to the degree of branching. Herein the ability for MIL‐160(Al) to separate these alkanes was first demonstrated by a series of fixed bed breakthrough experiments performed for powdered samples between 373 and 473 K with total isomer pressure up to 50 kPa (see Figures S12–S14 in the Supporting Information). **Figure** **2** shows that the HRON dibranched 22DMB and 23DMB elute first and they are completely separated from the LRON monobranched 3MP and 2MP while the LRON linear *n*‐C6 elutes much later. Notably, the sorption hierarchy of hexane isomers is similar to the boiling point order of the hydrocarbons: *n*‐C6 ≫ 2MP ≈ 3MP ≫ 23DMB > 22DMB. The shape of the breakthrough curves for each isomer clearly indicates that the excellent selectivity of MIL‐160(Al) is primarily based on equilibrium competition due to their steepness and roll‐up phenomenon, where the least adsorbed components (moving faster in the column) are pushed out from the adsorbent by the more strongly adsorbed ones (moving slower in the column), and therefore their concentration rises above the feed in a sequential manner. Figure 2 also shows the product mixture average RON at the outlet of the column. It can be read a maximum value around 99 at the time when only the dibranched isomers 22DMB and 23DMB already breakthrough the column completely separated from the other counterparts. Moreover, MIL‐160(Al) outperforms by far all current adsorbent materials in terms of productivity at a fixed RON of 92 (see Figure S16 in the Supporting Information) with an exceptional value of 1.15 mol dm^−3^ (see Section S4 in the Supporting Information) twice higher than that previously reported for the benchmark MOF (Fe_2_(BDP)_3_; 0.54 mol dm^−3^).^[^ ^19^ ^]^ Note the parent isostructural Al isopthalate (or 1,3‐benzene dicarboxylate) CAU‐10(Al) MOF^[^ ^29^ ^]^ exhibits a much lower level of performance (Figure S17, Supporting Information), its bulkier 6‐membered benzyl ring preventing an efficient packing of the isomers leading to a poor separation ability. This highlights the uniqueness of MIL‐160 framework for such a separation.

**Figure 3:**
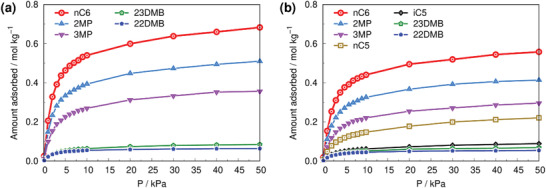
CBMC simulated adsorption isotherms of multicomponent C5‐C6 isomers in MIL‐160(Al) at 423 K. a) Adsorption isotherms of equimolar quinary mixture of hexane isomers. b) Adsorption isotherms of equimolar septenary mixture of all pentane and hexane isomers.

**Figure 4:**
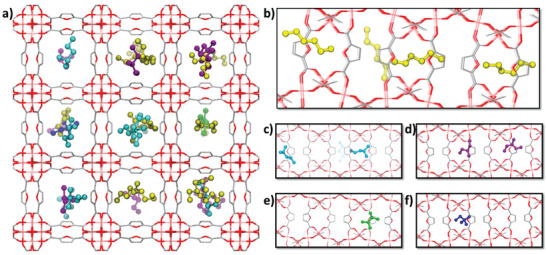
CBMC simulated microscopic view of the adsorbed C6 isomers in MIL‐160(Al). a) Illustration of the pore filling and spatial arrangement of all hexane isomers in the MOF pores at total isomer pressure of 100 kPa (*p_n_*_‐C6_ = *p*_2MP_ = *p*_3MP_ = *p*_23DMB_ = *p*_22DMB_ = 20 kPa) and *T* = 423 K. b–f) Illustrations of some representative molecular arrangements of *n*‐C6, 2MP, 3MP, 23DMB, and 22DMB, respectively, along the channel of MIL‐160 (Al). Color codes: *n*‐C6 (yellow spheres), 2MP (cyan spheres), 3MP (purple spheres), 23DMB (green spheres) and 22DMB (blue spheres), and MOF framework atoms: Al (pink), carbon (grey) and oxygen (red). Hydrogen atoms are omitted for clarity.

Configurational‐bias Monte Carlo (CBMC) simulations were further considered to shed light on the microscopic mechanism at the origin of the separation performance of MIL‐160(Al) (see details in the Experimental Section and Section S6 in the Supporting Information). CBMC calculations were first conducted for all single component C6 isomers and their equimolar binary mixtures *n*‐C6/2MP, 2MP/3MP, 2MP/23DMB, and 22DMB/23DMB. These simulations confirmed that the square channels of MIL‐160(Al) can accommodate all isomers and separate the linear to mono‐ and mono‐ to dibranched molecules, as observed experimentally (Figures S30–S34, Supporting Information). Moreover, Figure S30 (Supporting Information) reveals a good agreement between the experimental and simulated adsorption isotherms for all C6 isomers at 423 K that enabled to validate the host/guest force field parameters and the MIL‐160(Al) structure model. The sorption hierarchy simulated at 423 K for the quinary mixture, i.e., *n*‐C6 ≫ 2MP ≈ 3MP ≫ 23DMB > 22DMB (**Figure** **3**a) correlates well with the sequence of both MC‐derived Henry constants and DFT‐calculated host/guest binding energies for all isomers (Table S16, Supporting Information) and is in line with the breakthrough data collected at the same temperature. This observation clearly indicates that the separation is equilibrium‐based and not kinetically controlled. **Figure** **4**a illustrates the preferential sittings and conformations of each hexane isomer in the quinary mixture. The linear hexane molecules are found to be aligned both along and across the MIL‐160(Al) channel direction to favor interactions between the MOF pore wall and the largest fraction of the hexane skeleton (Figure 4b). Typically, in this arrangement *n*‐C6 interacts via its terminal ‐CH_3_ centers with two furan linkers and its –CH_2_ center with the adjacent side walls associated with standard van der Waals (vdw) dispersive interaction distances from 3.5 Å (see corresponding radial distribution functions in Figure S35 in the Supporting Information). In an optimal scenario (Figure S36, Supporting Information), the backbone of a *n*‐C6 aligns perpendicular to the channel in such a way that all CH_3_ and CH_2_ centers effectively interact with the MOF pore walls. This particular orientation maximizes the *n*‐C6/MIL‐160(Al) interactions from an interplay between the dimensionality of the MOF pore (5.8 Å) that fits with that of the linear *n*‐C6 molecule (4.3 Å), and the low sterically constrained five‐membered ring linkers that confer the linear molecules to establish close contacts with both furan ring and carboxylate groups. Thanks to their similar dimension and flexibility as *n*‐C6, the mono branched 2MP and 3MP isomers can be equally aligned across the MIL‐160(Al) channels (Figure 4c,d), interacting with opposite pore walls and multiple furan linkers as depicted in Figure S37 (Supporting Information) however associated with an adsorption affinity slightly lower than for *n*‐C6 (Table S16, Supporting Information). In sharp contrast, the more compact dibranched dimethyl butane isomers are shorter and not flexible enough to adapt similar optimal arrangements (Figure 4e,f), and only a small fraction of the carbon centers (1 or 2) effectively interact with the MOF pore walls (Figure S38, Supporting Information). This illustrates the weakening of the host/guest vdw interactions with increasing degree of branching and the low uptake of 22DMB and 23DMB in the quinary mixture.

In separation processes, shaped sorbent with the appropriate particle size and density is a practical prerequisite. The shaped particles, that usually contain a few wt% of an inorganic or polymeric binder, shall also maintain the sorption properties of the powder. Therefore, 2.0–3.35 mm shaped beads of MIL‐160(Al) were produced through a wet granulation process using an inorganic binder following a previously reported protocol.^[^ ^27^ ^]^ We further revealed that MIL‐160(Al) maintains its selectivity performance under similar working conditions with the use of these shaped beads, while only a minimal decrease in the 92 RON productivity was observed from 1.15 to 1.02 mol dm^−3^ resulting from the presence of binder incorporated in the shaped sample (Figure S23a, Supporting Information). This result is of importance for a further integration of this material in the industrial process.

### 2.2 MIL‐160(Al) for Separating Branched Hexane and Pentane Isomers into HRON and LRON Fractions

**Figure 5:**
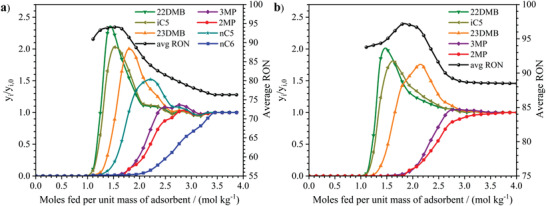
Separation of equimolar mixtures of pentane and hexane isomers by fixed bed adsorption with MIL‐160(Al) at 423 K. a) Separation of all pentane and hexane isomers. b) Separation of only branched pentane and hexane isomers. Experimental breakthrough data plotted as normalized molar fractions of each isomer (left *y*‐axis) and average real‐time RON (right *y*‐axis) as a function of moles fed per unit mass of pure of MIL‐160(Al) powder at a total isomers pressure of 50 kPa.

Since the feed of an actual TIP process contains both pentane and hexane isomers, fixed bed breakthrough experiments were further performed with a feed mixture containing all the pentane and hexane isomers in the powdered MIL‐160(Al) at 423 K and total isomer pressure of 50 kPa (**Figure** **5**a). The sorption hierarchy was found as follows: *n*‐C6 ≫ 2MP ≈ 3MP ≫ *n*‐C5 > 23DMB > i‐C5 ≈ 22DMB. Indeed, while the HRON i‐C5 and 22DMB elute early—practically together—unwantedly the LRON n‐C5 elutes later, almost simultaneously with the HRON 23DMB, leading to a substantial drop in the productivity to 0.69 mol dm^−3^ (vs 1.15 mol dm^−3^ for hexane isomers only) for a fixed RON of 92. This experimental sorption hierarchy was well captured by CBMC simulations, however with a lower simulated i‐C5 uptake as compared to the breakthrough data (Figure 3b). Interestingly, the sequence of both MC‐derived Henry constants and DFT‐calculated host/guest binding energies for all isomers (Table S16, Supporting Information) reproduces the experimental sorption hierarchy for the septenary mixtures that supports a thermodynamic‐driven separation for C5 and C6 isomers. The organization of all hexane molecules in the MIL‐160 channel remains the same as observed for the equimolar hexane isomers mixture (see Figure S39 in the Supporting Information). The conformations of the monobranched i‐C5 are rather similar to those of dibranched hexane isomers (see Figure S40 in the Supporting Information). In addition, linear *n*‐C5 molecules tend to adopt aligned configurations like *n*‐C6. However, its shorter length does not maximize the interactions with the pore walls, particularly in the conformation perpendicular to the channel, since only a fraction of its carbon sites establishes close contacts with the furan linkers (see Figure S41 in the Supporting Information).

Interestingly, if one removes the LRON normal paraffins (i.e., *n*‐C5 and *n*‐C6) from the feed mixture, a desired separation into two distinct fractions of HRON (23DMB > i‐C5 ≈ 22DMB) and LRON (2MP ≈ 3MP) paraffins (Figure 5b) is readily achieved. In this condition, at the early stage of the breakthrough when only the 22DMB/i‐C5/23DMB components elute from the column, a maximum RON of 97 is obtained. Importantly, MIL‐160(Al) exhibits a productivity of 2.56 mol dm^−3^ at an RON of 92 which becomes even higher at 373 K (3.92 mol dm^−3^). Noteworthy, this is the first experimental evidence that an adsorbent effectively fractionates pentane and hexane branched isomers into two distinct HRON and LRON fractions. CBMC simulations performed for the same equimolar quinary mixture of mono‐ and dibranched alkanes again confirmed this separation hierarchy driven by an equilibrium process (see Figure S42 in the Supporting Information).

### 2.3 Synergistic Action of MIL‐160(Al) and Zeolite 5A to Separate All Hexane and Pentane Isomers into Classes of HRON and LRON Fractions

**Figure 6:**
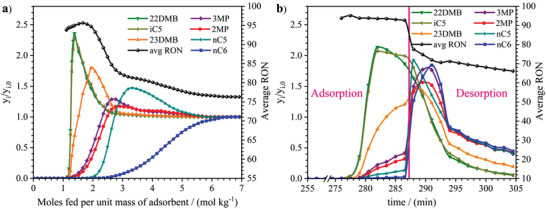
Separation of all pentane and hexane isomers by MIL‐160(Al) and zeolite 5A mixed‐bed adsorbent. a) Breakthrough data for an equimolar mixture feed in the mixed fixed bed at 423 K and total isomers pressure of 50 kPa. b) Cyclic steady state conditions of a PSA experiment in the same conditions. Adsorption, step I—pressurization and feed; desorption, step II—vacuum countercurrent depressurization followed by inert He purge. Total mass of adsorbents in the column for both experiments: 18.29 g (70 wt%) MIL‐160(Al)/binder‐free zeolite 5A (30 wt%)

As a next step, we deliberately chose to associate MIL‐160(Al) with the commercially available zeolite 5A—known for its unique size‐selective separation of linear/branched paraffins—to design a single adsorption bed able to feed all the pentane and hexane isomers and separate them into HRON and LRON fractions (Figure 1). The corresponding experimental breakthrough data collected for the mixed bed of 70 wt% MIL‐160(Al) (shaped beads of 2.0–3.35 mm) and 30 wt% zeolite 5A (binder‐free beads of 1.2–2.0 mm) at industrially relevant separation conditions (423 K and 50 kPa of total isomers pressure), demonstrates the desired discrimination between fractions of HRON (22DMB, 23DMB, and i‐C5), and LRON (*n*‐C6, *n*‐C5, 2MP, and 3MP) isomers. **Figure** **6**a reveals an ideal sorption hierarchy: *n*‐C6 ≫ *n*‐C5 ≫ 2MP ≈ 3MP ⋙ 23DMB > i‐C5 ≈ 22DMB associated with an excellent productivity of 1.14 mol dm^−3^ for an RON of 92. This ratio was selected in order to guarantee the complete displacement of the linear pentane nC5 wavefront forward the monobranched hexanes (2MP/3MP) to obtain a pure fraction of only HRON molecules (22DMB, iC5, and 23DMB). A comparison with the experiment performed at the same operating condition on a layered bed is reported in Figure S26 (Supporting Information).

A preliminary cyclic PSA experiment was then carried out with all pentane and hexane isomers mixture to prove that the MIL‐160(Al)/zeolite 5A pair can be effective to upgrade the actual TIP process under continuous cyclic adsorption operation. Figure 6b shows the cyclic steady state (CSS) of a simplified two‐step PSA experiment, i.e., i) pressurization and adsorption with feed and ii) vacuum countercurrent depressurization with inert He purge (desorption). This experiment revealed that at step I (adsorption), the mass front of the HRON 22DMB and i‐C5 has completely left the column, while the mass transfer front of 23DMB is concentrated at the edge of the column. The mass transfer front of the other LRON isomers mostly remains inside of the bed. Decisively, this PSA experiment confirms the viability of the novel mixed‐bed adsorbent to separate all pentane and hexane isomers in continuous cyclic operation into distinct HRON and LRON fractions.

## 3 Conclusion

This work reveals that a synergistic action of a mixed bed made by the robust bioderived MOF MIL‐160(Al) together with the commercially available zeolite 5A achieves an efficient separation of all pentane and hexane isomers according to the degree of branching, associated with valuable HRON and LRON fractions, leading to the complete fractionation of light naphtha (RON < 70) into an HRON hydrocarbon final product (RON > 92). The synergistic action of this mixed‐zeolite/MOF bed is required to prevent the elution of the linear LRON *n*‐C5 together with 23DMB in the HRON fraction if only a single bed of MOF MIL‐160(Al) is used. As a result, it was demonstrated that a spectacular separation of all pentane and hexane isomers under relevant industrial operating conditions is achieved with an exceptional ideal productivity of 1.14 mol dm^−3^. Through preliminary PSA experiments, this MOF/zeolite duo mixed bed was proven to operate under cyclic operating conditions and therefore be incorporated in the so‐called TIP process. As MIL‐160(Al) can be produced at multi‐kilogram scale with a predicted low‐cost industrial production^[^ ^25^ ^]^ while zeolite 5A is already commercially available, this gives the building block for further testing at pilot‐scale prior to envisaging industrial commercialization. This advanced staged recycling technology might not only provide the production of higher quality clean gasoline but also fills the gap in the current industrial adsorption separation science by combining two prominent classes of porous materials, paving the way for the design of new separative processes that will exploit MOFs and zeolites complementary features.

## 4 Experimental Section

### Breakthrough Experiments

Breakthrough experiments were performed in an experimental apparatus consisting of three main sections: 1) gas preparation, 2) adsorption, and 3) analytical section. In the gas preparation section (1), the carrier gas helium and the paraffins are introduced into the system by a mass flow controller (helium) and a syringe pump, respectively. Both went into a vaporizer before flowing into the adsorption column located inside the preparative gas chromatograph. The adsorption section (2) consists of a stainless‐steel column filled with the adsorbent materials in powder or/and shaped forms. The outlet stream of the fixed bed is directed to the analytical section (3) where a chromatograph equipped with a flame ionization detector (FID) analyzes its composition. Detailed information on the system's characteristics, experimental procedure and operating conditions are given in Sections S3 and S5 in the Supporting Information, respectively.

### PSA Experiments

The continuous cyclic studies of a two‐step PSA experiment with one single column are performed in an apparatus consisting also of three main sections: (i) gas preparation section; (ii) two‐step PSA adsorption column: step I ‐adsorption (pressurization with feed and feed at constant pressure), and step II ‐desorption (vacuumcountercurrrent depressurization with inert He purge); (iii) analytical section. The gas preparation (i) i) and analytical sections (iii) are the same used in the breakthrough experiments. The PSA adsorption column (2) is located inside a temperature‐controlled oven assembled with an automatic eight‐port valve. This automatic valve is preprogramed for switching between the adsorption (step I) and desorption (step II) over the entire experiment in predefined time intervals. Detailed information on the system's characteristics, experimental procedure and operating conditions are given in Sections S3 and S5 in the Supporting Information, respectively.

### Molecular Simulations

Configuration‐bias Monte Carlo (CBMC) simulations were performed to calculate the single component and mixture adsorption isotherms for pentane/hexane isomers in MIL‐160(Al) at 423 K. In these calculations, atoms of the MOF framework were described by single sites LJ parameters taken from the universal force field (UFF)^[^ ^30^ ^]^ while alkane molecules were modeled using the transferable potentials for phase equilibria united atom (TraPPE‐UA) models.^[^ ^31^ ^]^ Henry's constant (KH) for each of the pentane/hexane molecules was calculated using the revised Widom's test particle insertion method. Density functional theory calculations were equally performed to assess the host/guest binding energies for all single component pentane/hexane isomers. Details of these molecular simulations are described in Section S6 in the Supporting Information.

## Conflict of Interest

The authors declare no conflict of interest.

## Supporting information

Supporting Information

## Data Availability

The data that support the findings of this study are available from the corresponding author upon reasonable request.
