# Supplementary material for: Separation of Branched Alkanes Feeds by a Synergistic Action of Zeolite and Metal‐Organic Framework
Source: Adv Sci (Weinh). 2022 Jun 5;9(22):2201494. doi: 10.1002/advs.202201494 (PMC9353491; doi:10.1002/advs.202201494)
Supplement: Supplementary file 1 — Supporting Information [file ADVS-9-2201494-s001.pdf]

ADVANCED SCIENCE
Open Access

Supporting Information

for *Adv. Sci.*, DOI 10.1002/advs.202201494

Separation of Branched Alkanes Feeds by a Synergistic Action of Zeolite and Metal-Organic Framework

*Pedro F. Brântuas, Adriano Henrique, Mohammad Wahiduzzaman, Alexander von Wedelstedt, Tanmoy Maity, Alírio E. Rodrigues, Farid Nouar, U-Hwang Lee, Kyung-Ho Cho, Guillaume Maurin*, José A. C. Silva* and Christian Serre**

# Supporting Information

**Separation of Branched Alkanes Feeds by a Synergistic Action of Zeolite and Metal-Organic Framework**

*Pedro F. Brântuas, Adriano Henrique, Mohammad Wahiduzzaman, Alexander von Wedelstedt, Tanmoy Maity, Alírio E. Rodrigues, Farid Nouar, Guillaume Maurin*, José A. C. Silva*, and Christian Serre**

*Corresponding author.
E-mail: guillaume.maurin1@umontpellier.fr
E-mail: jsilva@ipb.pt
E-mail: christian.serre@espci.psl.eu

**TABLE OF CONTENTS**

**LIST OF FIGURES** .......... **3**
**LIST OF TABLES** .......... **7**
**1. PREPARATION OF MATERIALS** .......... **9**
1.1 Synthesis and characterization of powder MOF MIL-160(Al) .......... 9
*1.1.1. Powder X-Ray Diffraction* .......... *9*
*1.1.2. Infrared Spectroscopy* .......... *9*
*1.1.3. Thermogravimetric Analysis* .......... *10*
*1.1.4. Gas sorption study* .......... *10*
1.2 Synthesis and characterization of shaped MOF MIL-160(Al) .......... 10
1.3 Characterization of Zeolite 5A .......... 10
**2. SAMPLING OF MIL-160(AL)** .......... **10**
**3. EXPERIMENTAL SETUP AND PROCEDURE** .......... **11**
3.1 Experimental apparatus to measure breakthrough curves .......... 11
3.2 Experimental apparatus to perform continuous cyclic experiments .......... 12
3.3 Physical properties of the adsorption column and MFC´s and syringe characteristics .......... 14
3.4 Experimental procedure to measure breakthrough curves .......... 14
**4. PROCESS PERFORMANCE** .......... **15**
4.1 RON Calculations .......... 15
4.2 Productivity .......... 15
4.3 Adsorption Selectivity .......... 16
**5. EXPERIMENTAL MEASUREMENTS** .......... **16**
**6. COMPUTATIONAL DETAILS** .......... **20**
6.1 Configurational-bias Monte Carlo calculations .......... 20
6.2 DFT host-guest interaction energy calculations .......... 21
**REFERENCES** .......... **80**

**LIST OF FIGURES**

**Figure S1**. PXRD patterns of MIL-160(Al). ........................................................................ 22

**Figure S2**. FTIR spectra of MIL-160(Al) and organic linker FDCA. ................................................ 23

**Figure S3**. TGA analysis of MIL-160(Al). ........................................................................ 24

**Figure S4**. Nitrogen adsorption and desorption isotherms at 77 K on MIL-160(Al). ........................... 25

**Figure S5**. Pore size distribution and cumulative pore volume of MIL-160(Al). ................................ 26

**Figure S6**. Adsorbent materials in the shaped form: a) MIL-160(Al) beads (2.0 to 3.35 mm). b) Zeolite 5A binder-free beads (1.2 to 2.0 mm) ........................................................................ 27

**Figure S7**. Experimental procedure for agglomeration of powdered samples of MIL-160(Al). ............ 28

**Figure S8**. Schematic diagram of the experimental apparatus used to measure single and multicomponent breakthrough curves. (AC) adsorption column; (APC) advanced pneumatic control; (FID) flame ionization detector; (MFC) mass flow controller; (S/SP) split/splitless-injector; (SP) syringe pump; (TCD) thermal conductivity detector; (TL) transfer line; (V1) 4-way valve; (V2) and (V3) 6-way valve; (V4) 3-way valve. ........................................................................ 29

**Figure S9**. Schematic diagram of the experimental apparatus used to perform the continuous cyclic experiments: a) Gas preparation section for checking the paraffins vaporization; b) Pressurization with feed and adsorption; and c) Vacuum countercurrent depressurization with inert He purge desorption. Blue and green lines represent the paraffins (adsorption) and He purge (desorption) flow paths inside the system, respectively. ........................................................................ 30

**Figure S10**. Adsorption columns used in the sorption experiments. .................................................. 31

**Figure S11**. Experimental pure component adsorption equilibrium isotherms of hexane isomers on powder MIL-160(Al). a) 22DMB, b) 23DMB, c) 3MP, d) 2MP, and e) nC6 at 423K (circles) and 473 K (diamonds) ........................................................................ 32

**Figure S12**. Experimental multicomponent breakthrough curves for an equimolar quinary mixture of hexane isomers on powder MIL-160 at 373 K and a) 10 kPa, b) 25.0 kPa, and c) 50kPa. ..................... 33

**Figure S13**. Experimental multicomponent breakthrough curves for an equimolar quinary mixture of hexane isomers on powder MIL-160 at 423 K and a) 10 kPa and b) 25 kPa. ........................................ 34

**Figure S14**. Experimental multicomponent breakthrough curves for an equimolar quinary mixture of hexane isomers on powder MIL-160 at 473 K and a) 10 kPa, b) 25.0 kPa, and c) 50kPa. ..................... 35

**Figure S15**. Experimental quinary adsorption equilibrium isotherms for an equimolar mixture of hexane isomers on powder MIL-160 at (a) 373 K, (b) 423 K, and (c) 473 K. ....................................................36

**Figure S16**. Comparison of the 92 RON productivities for separating equimolar quinary mixtures of hexane isomers (nC6/2MP/3MP/23DMB/22DMB) using a variety of materials under similar working conditions. The blue column represents the experimental data obtained on MIL-160(Al) at a total hydrocarbon pressure of 50 kPa ($p_{n\text{-}C6} = p_{2MP} = p_{3MP} = p_{23DMB} = p_{22DMB} = 10$ kPa) and temperature of 423 K, while all other columns in orange represents the data reported by Herm et al.,[5] which are based on CBMC simulations performed at a total hydrocarbon pressure of 100 kPa ($p_{n\text{-}C6} = p_{2MP} = p_{3MP} = p_{23DMB} = p_{22DMB} = 20$ kPa) and temperature of 433 K. ..........................................................................37

**Figure S17**. Experimental multicomponent breakthrough curves for an equimolar quinary mixture of hexane isomers on powder CAU-10 at 423 K and 50kPa. .....................................................................38

**Figure S18**. Experimental multicomponent breakthrough curves for an equimolar septenary mixture of pentane and hexane isomers on powder MIL-160 at 50 kPa and a) 373 K and b) 473 K. ......................39

**Figure S19**. Experimental multicomponent breakthrough curves for an equimolar quinary mixture of branched pentane and hexane isomers on powder MIL-160 at 50 kPa and a) 373 K and b) 473 K. ......40

**Figure S20**. Experimental pure component adsorption equilibrium isotherms of pentane and hexane isomers on shaped MIL-160(Al). a) iC5, b) nC5, c) 22DMB, d) 23DMB, e) 3MP, f) 3MP, and g) nC6 at 373 K (squares), 423K (circles), and 473 K (diamonds). ......................................................................41

**Figure S21**. Experimental multicomponent breakthrough curves for an equimolar binary mixture of hexane isomers on shaped MIL-160 at 423 K and 50 kPa. a) 22DMB/23DMB, b) 23DMB/2MP, c) 3MP/2MP, and d) 2MP/nC6. ..........................................................................................................42

**Figure S22**. Experimental multicomponent breakthrough curves for an equimolar ternary mixture of hexane isomers on shaped MIL-160 at 423 K and 50 kPa. a) 22DMB/2MP/nC6 and b) 23DMB/2MP/nC6 ..........................................................................................................................................43

**Figure S23**. Experimental multicomponent breakthrough curves for an equimolar quinary mixture of hexane isomers on shaped MIL-160 at 50 kPa and a) 423 K and b) 473 K. ..........................................44

**Figure S24**. Experimental multicomponent breakthrough curves for a septenary mixture of pentane and hexane isomers on shaped MIL-160 at 423 K and 50 kPa. The concentration used is equal to one reported by Holcombe et al.,[6] which corresponds to the composition of the product stream from an isomerization reactor. ..........................................................................................................................45

**Figure S25**. Experimental multicomponent breakthrough curves for a quinary mixture of pentane and hexane isomers on shaped MIL-160 at 423 K and 50 kPa. The concentration used is equal to one reported

by Holcombe et al.,[6] which corresponds to the composition of the product stream from an isomerization reactor, considering the branched isomers as the only paraffins in the mixture. ....................................46

**Figure S26**. Experimental multicomponent breakthrough curves for an equimolar septenary mixture of pentane and hexane isomers on a bed of shaped MOF MIL-160(Al) (70 wt%) and binder-free beads Zeolite 5A (30 wt%) at 423 K and 50 kPa. a) Mixed bed and b) Layered bed (mixture passing through the zeolite 5A packing first). ....................47

**Figure S27**. Experimental multicomponent breakthrough curves for an equimolar septenary mixture of hexane isomers on a mixed bed of shaped MOF MIL-160(Al) (70 wt%) and binder-free beads Zeolite 5A (30 wt%) at 473 K and 50 kPa. ....................48

**Figure S28**. Experimental preliminary PSA test for an equimolar septenary mixture of pentane and hexane isomers on a mixed bed of shaped MOF MIL-160(Al) (70%) and binder-free beads Zeolite 5A (30 wt%) at 473 K and 50.0 kPa. Steady-state effluent concentration. ....................49

**Figure S29**. Molecular representation of the pentane/hexane isomers. Implicit hydrogen atoms are lumped onto the neighboring carbons and described with pseudo atoms $CH_3$, $CH_2$, CH, and C according to TraPPE-UA potentials. ....................50

**Figure S30**. Experimental and simulated single component adsorption isotherms of hexane isomers on MIL-160(Al) for the (a) pristine and (b) and 5° linker rotation obtained at $T$ = 423 K. Markers represent the experimental data, and the continuous lines represent the CBMC simulations. ....................51

**Figure S31**. CBMC simulated co-adsorption isotherms of equimolar *n*-C6/2MP binary mixture for the MIL-160(Al) with 5° tilted linkers at $T$ = 423 K (left panel) and a snapshot of the adsorbed *n*-C6 (green spheres) and 2MP (purple spheres) molecules at $P$ = 10 kPa within the channel of the MOF (right panel). ....................52

**Figure S32**. CBMC simulated co-adsorption isotherms of equimolar 2MP/3MP binary mixture for the MIL-160(Al) with 5° tilted linkers at $T$ = 423 K (left panel) and a snapshot of the adsorbed 2MP (green spheres) and 3MP (purple spheres) molecules at $P$ = 10 kPa within the channel of the MOF (right panel). ....................53

**Figure S33**. CBMC simulated co-adsorption isotherms of equimolar 2MP/23DMB binary mixture for the MIL-160(Al) with 5° tilted linkers at $T$ = 423 K (left panel) and a snapshot of the adsorbed 2MP (green spheres) and 23DMB (purple spheres) molecules at $P$ = 10 kPa within the channel of the MOF (right panel). ....................54

**Figure S34**. CBMC simulated co-adsorption isotherms of equimolar 22DMB/23DMB binary mixture for the MIL-160(Al) with 5° tilted linkers at $T$ = 423 K (left panel) and a snapshot of the adsorbed 22DMB

(green spheres) and 23DMB (purple spheres) molecules at *P* = 10 kPa within the channel of the MOF (right panel).......................................................................................................................55

**Figure S35**. Intermolecular radial pair distribution functions of the (a) $CH_3$(UA), (b) $CH_2$(UA), (c) CH(UA), and (d) C of the hexane isomers with respect to MIL-160(Al) framework atoms in the vicinity of the pore channel calculated for an equimolar quinary mixture at *T* = 423 K and *P* = 10 kPa. ...........56

**Figure S36**. Illustration of a typical spatial arrangement and van der Waals close contacts (<3.5 Å) of an *n*-C6 molecule with the MIL-160(Al) pore walls taken from a representative adsorption snapshot derived from CBMC simulation performed for an equimolar quinary mixture of all hexane isomers at *T* = 423 K and *P* = 20kPa. .......................................................................................................................57

**Figure S37**. Illustration of a typical spatial arrangement and van der Waals close contacts (<3.5 Å) of a 2MP molecule with the MIL-160(Al) pore walls taken from a representative adsorption snapshot derived from CBMC simulation performed for an equimolar quinary mixture of all hexane isomers at *T* = 423 K and *P* = 20 kPa. .....................................................................................................................58

**Figure S38**. Illustration of a typical spatial arrangement and van der Waals close contacts (<3.5 Å) of a 23DMB molecule with the MIL-160(Al) pore walls taken from a representative adsorption snapshot derived from CBMC simulation performed for an equimolar quinary mixture of all hexane isomers at *T* = 423 K and *P* = 20 kPa. ...........................................................................................................59

**Figure S39**. Illustration of CBMC simulated pore filling and spatial arrangement of all pentane and hexane isomers in the MOF channel obtained for an equimolar septenary mixture of C5/6 isomers at *T* = 423 K and *P* = 20 kPa. Color codes: *n*-C5 (orange spheres), iC5 (black spheres), *n*-C6 (yellow spheres), 2MP (cyan spheres), 3MP (purple spheres), 23DMB (green spheres) and 22DMB (blue spheres), MOF framework atoms: Al (pink), Carbon (grey) and Oxygen (red), hydrogen atoms are omitted for clarity. ..............................................................................................................................................60

**Figure S40**. Illustration of a typical spatial arrangement and van der Waals close contacts (<3.5 Å) of an iC5 molecule with the MIL-160(Al) pore walls taken from a representative adsorption snapshot derived from CBMC simulation performed for an equimolar septenary mixture of all C5 and C6 isomers at *T* = 423 K and *P* = 20 kPa. .........................................................................................................61

**Figure S41**. Illustration of a typical spatial arrangement and van der Waals close contacts (<3.5 Å) of an *n*-C5 molecule with the MIL-160(Al) pore walls taken from a representative adsorption snapshot derived from CBMC simulation performed for an equimolar septenary mixture of all C5 and C6 isomers at *T* = 423 K and *P* = 20 kPa. .........................................................................................................62

**Figure S42**. CBMC simulated co-adsorption isotherms of equimolar iC5/2MP/3MP/23DMB/22DMB mixture for the MIL-160(Al) with 5° tilted linkers at *T* = 423 K. .........................................................63

**LIST OF TABLES**

**Table S1**. Textural properties of MIL-160(Al). ........................................................................64

**Table S2**. Column, MFC´s, and syringe characteristics for the experimental studies. ..........................65

**Table S3**. Molecular dimensions and research octane numbers of pentane and hexane isomers. ..........66

**Table S4**. Experimental conditions to measure pure component breakthrough curves of hexane isomers on powder MIL-160(Al). ........................................................................................................67

**Table S5**. Experimental conditions to measure multicomponent breakthrough curves for an equimolar quinary mixture of hexane isomers on powder MIL-160(Al). ..............................................................68

**Table S6**. Experimental conditions to measure multicomponent breakthrough curves for an equimolar quinary mixture of hexane isomers on powder CAU-10(Al). ................................................................69

**Table S7**. Experimental conditions to measure multicomponent breakthrough curves for an equimolar quinary/septenary mixture of pentane and hexane isomers on powder MIL-160(Al)............................70

**Table S8**. Experimental conditions to measure pure component breakthrough curves of pentane and hexane isomers on shaped MIL-160(Al). ..........................................................................................71

**Table S9**. Experimental conditions to measure multicomponent breakthrough curves for an equimolar binary/ternary mixture of hexane isomers on shaped MIL-160(Al)......................................................72

**Table S10**. Experimental conditions to measure multicomponent breakthrough curves for an equimolar quinary mixture of hexane isomers on shaped MIL-160(Al). ................................................................73

**Table S11**. Experimental conditions to measure multicomponent breakthrough curves for a septenary mixture of pentane and hexane isomers on shaped MIL-160(Al) with an isomerization concentration.[6] ..........................................................................................................................................74

**Table S12**. Experimental conditions to measure multicomponent breakthrough curves for an equimolar septenary mixture of pentane and hexane isomers in a mixed bed of shaped MOF MIL-160(Al) (70 wt%) and binder-free beads Zeolite 5A (30 wt%)..........................................................................................75

**Table S13**. Experimental conditions for preliminary PSA tests for an equimolar septenary mixture of pentane and hexane isomers in a mixed bed of shaped MOF MIL-160(Al) (70 wt%) and binder-free beads Zeolite 5A (30 wt%). ..........................................................................................................76

**Table S14**. Intermolecular LJ-potential parameters for the pentane and hexane isomers. .....................77

**Table S15**. Intermolecular LJ Potential parameters for the MIL-160(Al) framework atoms. ................78

**Table S16**. DFT-calculated host-guest binding energies and CBMC calculated Henry constants ($K_H$) for the adsorbed C5-C6 alkane isomers in the MIL-160(Al). ........................................................................79

# 1. PREPARATION OF MATERIALS

## 1.1 Synthesis and characterization of powder MOF MIL-160(Al)

The synthesis parameters for MIL-160(Al) were adapted from previously reported conditions:[1] 1.2 g (7.5 mmol) of 2-furandicarboxylic acid and 1.17 g (7.5 mmol) of $Al(OH)(CH_3COO)_2$ were mixed with 15 mL of distilled water and then subject to reflux at 383 K for 24 hours. After cooling to room temperature, the solid material was collected by filtration, washed with ethanol, and dried in a vacuum oven.

For the characterization of MIL-160(Al), several physicochemical measurements were performed: powder x-ray diffraction (PXRD), Fourier transformed infrared spectroscopy (FTIR), thermogravimetric analysis (TGA), and gas adsorption study to understand its porous nature. The PXRD patterns of the sample were recorded with a Bruker D8 ADVANCE diffractometer using Cu Kα radiation (λ = 1.5418 Å). FTIR spectra were measured using the ATR technique on a Perkin-Elmer Spectrum 100 spectrometer. Thermogravimetric analysis was performed on a Mettler Toledo TGA2 unit under an $O_2$ atmosphere at a 3 K $min^{-1}$ rate. Nitrogen adsorption and desorption isotherms at 77 K and pressure range of 0 – $10^5$ Pa were measured using a micromeritics TriStar II Plus gas sorption system. A ~ 50 mg material sample was introduced into an analysis tube and was evacuated under dynamic vacuum (for 6 h) at the desired temperature (423 K) using a micromeritics Smart VacPrep high vacuum pump. The Horvath-Kawazoe method (Slit Pore Geometry) was applied to evaluate the pore sizes distribution of the samples.

### *1.1.1.Powder X-Ray Diffraction*

The MIL-160(Al) PXRD patterns are illustrated in **Figure S1**. PXRD patterns of MIL-160(Al)., showing that the sample synthesized corresponds to MIL-160(Al). Comparison of synthesized materials (blue) with calculated powder pattern (red, extract from .cif) suggest that the purity and quality of MIL-160(Al) are very good and also match with the reported pattern.[1]

### *1.1.2.Infrared Spectroscopy*

The FTIR spectra of MIL-160(Al) and organic linker (FDCA) are shown in **Figure S2**. Both spectra reveal that there is no presence of unreacted acid linker in the MOF sample.

*1.1.3.Thermogravimetric Analysis*

**Figure S3** reveals the TGA analysis under the $O_2$ atmosphere of MIL-160(Al) (heating rate of 3 K $min^{-1}$). The measurements show an initial 27% mass loss up to 373 K. This corresponds to adsorbed solvents and water molecules within the pores of the MOF. After that, the material is stable up to 573 K and then degrades. These results are well-matched with the reported curve.[1]

*1.1.4.Gas sorption study*

Gas sorption measurements were undertaken to understand the porous nature of MIL-160(Al) synthesized material. **Figure S4** shows the $N_2$ adsorption and desorption isotherms at 77 K on MIL-160(Al), while **Figure S5** exhibits the pore size distribution and cumulative pore volume. As can be seen, the $N_2$ adsorption isotherms are of type I according to the IUPAC classification,[2] which is a signature characteristic of microporous materials. Brunauer Emmett Teller (BET) model was used for the specific surface area calculation, being the data reported in **Table S1**, together with the average pore size diameter and the maximum pore volume.

1.2 Synthesis and characterization of shaped MOF MIL-160(Al)

The shaping procedure of powdered MIL-160(Al) is detailed in the patent WO 2016/186454 A1 filled by the Korea Research Institute of Chemical Technology (KRICT),[3] with the result being shown in **Figure S6**a.

1.3 Characterization of Zeolite 5A

The binder-free beads of zeolite 5A were supplied by Chemiewerk Bad Koestritz GmbH (Germany), consisting of spherical particles with a diameter size ranging from 1.2 to 2.0 mm, as seen in **Figure S6**b.

## 2. SAMPLING OF MIL-160(AL)

Prior to screening studies, the MIL-160(Al) material in powdered form was transformed into small agglomerates to minimize diffusion issues. The experimental procedure is described in **Figure S7**. First, the powdered particles were compacted into tablets in a manual hydraulic press machine (GS15011,

Specac); the pressure applied was 6.5 kg $mm^{-2}$. Subsequently, the tablets were broken into small fragments and sieved to produce a known particle size distribution of around 2.5 mm.

## 3. EXPERIMENTAL SETUP AND PROCEDURE

### 3.1 Experimental apparatus to measure breakthrough curves

The equilibrium adsorption of pentane and hexane isomers in MIL-160(Al) were assessed in an apparatus developed to measure fixed-bed single and multicomponent breakthrough curves in the vapor phase, as shown in **Figure S8**. It mainly comprises two gas chromatographs (GC´s, YL 6500, YL Instruments Co., Ltd.), one being preparative and the other being analytic. Both GC´s have a shockproof design and stable structure against oven temperature changes. The experimental setup consists of three main sections: (*i*) gas preparation, (*ii*) adsorption, and (*iii*) analytical section.

In the gas preparation section, the carrier gas helium (99.9996%, Linde) and the paraffins are introduced into the system. Helium is used as a carrier gas due to its inertness and enters the system in four different streams: the lines (1), (2), make-up, and (8). The first three lines are directed to the preparative chromatograph, and line (8) is sent to the analytical part. The lines (1) and (2) are monitored by the mass flow controller (MFC, Alicat Scientific) while the make-up and line (8) pass through an advanced pneumatic control system (APC). The hydrocarbon stream is continuously introduced (in liquid phase) with a syringe pump (SP, Legato® 100, KD Scientific) in the carrier gas flowing in the line (1) to run into the inner heating region in a packed column inlet located in the preparative chromatograph.

The adsorption section consists of a stainless-steel column filled with the adsorbent material and operated inside the preparative chromatograph oven. The stream from the packed column inlet runs into the switching 4-ports (V1) and 6-ports valve (V2, VICI, Valco Instruments Co.), bypassing the adsorption column. Simultaneously, the pure helium flowing in line 2, which also passes through these two valves, goes to the adsorption column via line 5. The column bypass is incorporated to check if the paraffins vaporization is stable with a constant concentration in the Thermal Conductivity Detector (TCD), while the make-up line is used to dilute the signal of the paraffins. When the concentration reaches a constant signal, the experiment can be started. Therefore, the 3-way ball valve (V4, SS-41GXS1, Swagelok) is directed to vent manually, and when the inert baseline is reached in the TCD (pure helium signal), the V1 valve is actuated, allowing the paraffins to run through the adsorption column. The transfer line which connects the preparative to the analytic GC is thermally controlled by a microcomputer-based digital temperature indicating controller (TL, ACS-13A, Shinko) to keep the temperature stable and avoid any condensation of the gases from the preparative to the analytic chromatograph.

In the analytical section, used only for multicomponent experiments, the effluent flowing in the transfer line runs into a sampling 6-ports valve (V3, VICI, Valco Instruments Co.) inside the analytic chromatograph. In a pre-defined time interval, this valve sends an aliquot of gas to a capillary column (CC, with a 15 m length, 0.1 mm outside diameter, and 0.1 μm poly(dimethyl sulfoxane) coating, Supelco), via line (9) by the pure helium stream coming from the line (8). Before entering the capillary column, the aliquot of gas passes through a split/splitless (S/SP) injector. The capillary column is immersed in an ice-water bath to separate the component peaks better. Finally, the capillary column output goes to the Flame Ionization Detector (FID) to be analyzed. This detector uses air (reconstituted air K with a concentration of 80% $N_2$ and 20% $O_2$, Linde) and hydrogen (99.9999%, Alpha Gaz) to produce its flame.

When the experiment finishes, i.e., the saturation state is reached, the paraffins injection by the syringe pump is cut, and the V1 valve is actuated. Then, the carrier gas flowing through line (2) performs the packed column desorption. After that, another experiment can be performed. Before the first RUN, the packed column was activated for 12 h at 473 K under a pure helium flow. The helium lines corresponding to the reference side of TCD and split flow for the split/splitless injector (both controlled by the APC´s modules) were omitted from **Figure S8** for clarity.

The MFC´s, SP, (V1, V2, and V3) VICI valves, and TF are connected to a laboratory power supply (230V AC) with a USB control module, which sends their digital data to a personal computer (PC) where they are automated. Oven temperatures, APC modules, and TCD and FID detectors are controlled and recorded using the YL-Clarity data acquisition software (YL Instruments Co., Ltd.), an intuitive chromatography data system with a user-friendly interface.

For the whole adsorption study, the hexanes isomers 22DMB/23DMB/nC6 were supplied by Sigma Aldrich (> 99, 98, and 99%, respectively), 2MP by Alfa Aesar (> 99%), and 3MP by Acrōs Organics (> 99%); while the pentane isomers nC5 by Riedel-de Haën (> 99%) and the iC5 by Fluka (> 99%).

## 3.2 Experimental apparatus to perform continuous cyclic experiments

The continuous cyclic studies of preliminary PSA experiments on the zeolite 5A/MIL-160(Al) mixed-bed were performed on the apparatus shown in **Figure S9**. The equipment was designed with the main characteristics of versatility and a wide range of operating conditions. As it can be seen, one adapted the experimental setup previously showed to measure the adsorption equilibrium of pentane and hexane isomers (**Figure S8**) to perform tests at a larger pilot level. The main differences consist on:

1 - A new oven (FD 53, Binder) assembled with an 8-ports valve (V3, VICI, Valco Instruments Co.) is incorporated in the system, in which the adsorption column is now operated. The oven is equipped with forced convection to promote a homogeneous temperature distribution, keeping the column near-isothermal conditions. The V3 valve is used for switching between the adsorption and desorption steps of the cyclic experiment. This valve is also automated;

2 - The arrangement of the valves from the preparative chromatography was also modified. The V1 valve was rearranged to switch between the by-pass of the adsorption column for checking the paraffins vaporization stability on the TCD detector (preliminary step of the experiment) and sending the hydrocarbon mixture to the column oven. The V2 valve, adapted to act as a 4-ports valve, is now in charge of directing the output of the packed column to the TCD detector and posterior analytical stage. All the lines between the column oven and the preparative GC are heat traced and insulated with ceramic wool and aluminum tape. The temperature of these lines is controlled by a temperature regulator (TR, HT-MC1, Horst GmbH);

3 - Two electronic high-speed pressure transducers (PT, PXM409-010BAUSBH, OMEGA) were installed in the packed column effluent lines (adsorption and desorption) to record the history of the pressure behavior during the pressurization and depressurization stages. The PT´s are resistant to shock and vibrations and connects directly to the PC via USB, being the digital data recorded by the free OMEGA PC software;

4 - A diaphragm vacuum pump (VP, N810 Laboport, KNF) was also installed on the system to perform the depressurization of the packed column in the continuous cyclic experiments. Moreover, a flow metering valve (V4, SS-SS1-VH, Swagelok) was coupled to the vacuum pump to control the pressure in which the depressurization set point is stated. This valve operates manually.

The homemade apparatus developed to perform cyclic studies of a simplified 2 step PSA experiment with one single column is divided into three main sections: (*i*) gas preparation, (*ii*) adsorption (pressurization with feed + analysis), and (*iii*) desorption (vacuum countercurrent depressurization with inert He purge).

The gas preparation section is similar to the ones for the screening studies and is represented in **Figure S9**a. The carrier gas helium and the paraffins are introduced into the system, and the stability of this mixture vaporization should be checked. When the inert gas fills all the lines, the syringe pump starts to continuously introduce the hydrocarbons (in liquid phase) in the carrier gas from MFC to run through the vaporizer in the preparative chromatograph. Then, the vaporized mixture runs into the V1 valve, mixes with the make-up line, and goes directly to the TCD detector. Simultaneously, the pure helium from the other MFC runs into the V3 valve, packed column, vacuum pump, V2 valve and goes to the preparative chromatograph's vent. When the vaporized mixture reaches a constant concentration, the V1

valve is actuated, and the mixture goes to the column oven, where the adsorption column will be operated and leaves the system by the vent line. A manual bubble flowmeter is used to check that the mixture is coming out from the system.

As soon as the paraffins mixture fills the line between the preparative and column oven (at this time, the inert baseline in the TCD (pure helium signal) is already reached), the adsorption section takes place, and the experiment can be started (**Figure S9**b). Therefore, the V3 valve is actuated. The adsorption column, mainly filled with He, is pressurized with the feed mixture. The pressure in the column rises from vacuum to atmospheric pressure. The feed mixture passes through the packed column, and an HRON enriched fraction is withdrawn through the product end. The column output goes back to the preparative chromatography, runs into the V2 valve, which is already in the position to send the mixture to the TCD, and flows through the transfer line to the analytical chromatography to evaluate its composition. The analytical procedure is the same as the ones described in section 0.

When the adsorption step finishes, the desorption stage occurs (Figure S9**Erro! Fonte de referência não encontrada.**c). For that, both V3 and V2 valves are actuated. The column is then countercurrent depressurized with the vacuum pump and purged with a He stream. The effluent, mainly LRON molecules, is sent to the analytical section for chromatographic evaluation. Once the desorption time is up, another cycle can be started until reaching the cyclic steady state.

### 3.3 Physical properties of the adsorption column and MFC´s and syringe characteristics

The sorption studies were performed with the MIL-160(Al) material in different forms and scales, being the experiments conducted on the agglomerates (originated from powdered form) in milligram scale, and on the shaped beads at gram scale. Consequently, the physical properties of the adsorption column and the specifications of MFC´s controllers and syringes were chosen according to the operating conditions, as summarized in **Table S2**. The three different adsorption columns used in this work can be seen in **Figure S10**.

### 3.4 Experimental procedure to measure breakthrough curves

The experimental procedure to obtain single and multicomponent breakthrough curves consists of continuously measuring the concentration profile of the hydrocarbon species as a function of time at the outlet of the adsorption column. Therefore, the packed bed is operated by introducing at the inlet the feed containing the adsorbable species diluted in a helium stream at a fixed total hydrocarbon pressure and temperature.

The number of moles of each hydrocarbon retained in the packed bed at equilibrium conditions, $q_{exp,i}$ $(mol.kg^{-1})$, often called as equilibrium loading (also amount adsorbed) is obtained by integrating the concentration profiles (molar rate history) of the complete breakthrough curves, as follows:

$$q_{exp,i} = \frac{1}{m_{ads}}\left(F_{0,i}t_n - \int_0^{t_s} F_i dt - \varepsilon_b V_c C_{0,i}\right) \tag{S1}$$

where $m_{ads}$ $(kg)$ is the mass of adsorbent packed in the column, $F_{0,i}$ $and$ $F_i$ $(mol.s^{-1})$ are the molar flow rate of hydrocarbon species at the feed inlet and outlet of the column, respectively, $t_s$ $(s)$ is the saturation time, $\varepsilon_b$ is the bed porosity, $V_c$ $(m^3)$ is the volume of the column, and $C_{0,i}$ $(mol.m^{-3})$ is the feed gas concentration of the hydrocarbon species.

Complete information about the integrating procedure has already been reported elsewhere.[4]

## 4. PROCESS PERFORMANCE

### 4.1 RON Calculations

The real-time (instantaneous) RON of the hydrocarbon mixture leaving the adsorption column is calculated from the pure component $RON_i$ values summarized in **Table S3** averaged over the exit gas composition, as follows:

$$RON = \frac{\sum_{i=1}^{n}(y_i \times RON_i)}{\sum_{i=1}^{n} y_i} \tag{S2}$$

where $y_i$ is the molar fraction of the isomer $i$ at the column outlet and $n$ is the number of paraffins in the mixture.

### 4.2 Productivity

A realistic comparison of relevance to industrial applications can be performed by comparing the adsorbent productivity at an averaged 92 RON, i.e., the number of moles product obtained per cubic decimeter of adsorbent in a packed bed adsorber.[5] To calculate the productivity, we defined that the RON value of the entire gas mixture, thus extracted, is averaged to 92. Therefore, the real-time RON of the hydrocarbon mixture leaving the adsorption column (from Equation S1) is averaged for the appropriate time interval until the desired RON product is recovered. The volumetric productivity, $P_{ads}$ $(mol.dm^{-3})$, is then obtained by multiplying the gravimetric productivity (calculated through a

material balance over the adsorber) by the framework density of the material, $\rho_{ads}$ $(kg.dm^{-3})$, as follows:

$$P_{ads} = \left\{\sum_{i=i}^{n}\left[\frac{1}{m_{ads}}\left(\int_{0}^{t_{RON92}} F_i dt - \varepsilon_b V_c C_{0,i}\right)\right]\right\}.\rho_{ads} \quad \text{(S3)}$$

where $t_{RON92}$ $(s)$ is the appropriate time interval in which the averaged 92 RON value is obtained.

The framework density of MIL-160(Al) in the powdered state and the form of shaped beads is equal to 1.12 and 0.903 $kg.dm^{-3}$, respectively. For the binder-free beads of Zeolite 5A, the value is equal to 1.17 $kg.dm^{-3}$.

### 4.3 Adsorption Selectivity

The adsorption selectivities ($S_{ads}$) from multicomponent experiments performed with equimolar mixtures were measured by the ratio of the loadings between LRON paraffins over the HRON ones according to the following adapted[5] equation:

$$S_{ads} = \frac{q_{nC5} + q_{nC6} + q_{2MP} + q_{3MP}}{q_{iC5} + q_{23DMB} + q_{22DMB}} \quad \text{(S4)}$$

This definition is based on adsorbent materials that show a normal adsorption hierarch, wherein the adsorption strength of isomers species decreases as the degree of branching increases.

## 5. EXPERIMENTAL MEASUREMENTS

The adsorption behavior of pentane and hexane isomers in a fixed bed of MIL-160(Al) was investigated through a set of single and competitive breakthrough curves (binary, ternary, quinary, and septenary) performed at different temperatures and total hydrocarbon pressures relevant to the industrial separation. Helium was used to set up a total pressure in the column of 101.3 kPa. The breakthrough curves are expressed in terms of the ratio of normalized molar $y_i/y_{i0}$ as a function of the total molar amount of paraffins fed to the column per unit mass adsorbent.

**Figure S11** shows the pure component adsorption equilibrium isotherms of hexane isomers on powder MIL-160(Al) at 423 and 473 K and hydrocarbon pressures ranging from 5 to 50 kPa. **Table S4** summarizes the experimental conditions applied to measure the pure component breakthrough curves and the equilibrium loadings.

**Figure S12**,**Figure S13Figure S13**, and **Figure S14** show the breakthrough curves for an equimolar quinary mixture of hexane isomers on powder MIL-160(Al) at 373, 423, and 473 K, respectively. The effect of the total hydrocarbon pressure is shown in each figure: a) 10, b) 25, and c) 50 kPa. The experiment performed at 423 K and 50 kPa is presented in the main text (Figure 2). Complete information on the experimental fixed bed runs performed, the equilibrium loadings, and selectivities are given in **Table S5**. These results emphasize that MIL-160(Al) can efficiently fractionate the hexane isomers according to the degree of branching linear (nC6) > mono-branched (2MP, 3MP) > di-branched (23DMB, 22DMB) isomers. The calculated selectivities slightly decrease with temperature, varying from 6.3 at 373 K to 4.0 at 473 K, indicating that, at lower temperatures where the adsorption mixture loadings are enhanced, the equilibrium competition between the alkane isomers for adsorption sites is favored, resulting in a higher degree of separation. For instance, at 373 K (Figure S12), this competition leads to rises in concentration more than 200 % above that on the initial feed for the di-branched hexanes. As the temperature increases, these overshoots in concentration become smaller since the adsorption mixture loading decreases (Figure S13 and**Figure S14**). Nevertheless, the breakthrough curves specify that the separation by classes happens at all temperatures, meaning that the temperature effect is practically the same for all C6 isomers, in which their elution time was reduced, but the difference between them does not significantly change. Concerning the total hydrocarbon pressure effect, the selectivities remained practically constant for the considered range. The corresponding quinary adsorption equilibrium isotherms obtained from these multicomponent experiments are shown in **Figure S15.**

**Figure S16** shows a comparison of the 92 RON productivity for the separation of equimolar quinary mixtures of hexane isomers (nC6/2MP/3MP/22DMB/23DMB) on the MIL-160(Al) with a variety of other adsorbent materials at similar working conditions. The blue column represents the experimental data obtained on MIL-160(Al) at a total isomer pressure of 50 kPa ($p_{n\text{-}C6} = p_{2MP} = p_{3MP} = p_{23DMB} = p_{22DMB} = 10$ kPa) and temperature of 423 K. All the other columns in orange represents the data reported by Herm et al.[5], which are based on CBMC simulations performed at a total isomer pressure of 100 kPa ($p_{n\text{-}C6} = p_{2MP} = p_{3MP} = p_{23DMB} = p_{22DMB} = 20$ kPa) and temperature of 433 K. The productivity calculated for MIL-160(Al) (1.15 mol.dm$^{-3}$) is significantly higher than the ones previously reported, being more than two times higher than that of MOF $Fe_2(BDP)_3$ (0.54 mol dm$^{-3}$), which, until now, has been considered one of the most suitable materials for separating hexane isomers.

**Figure S17** shows the multicomponent breakthrough curve for an equimolar quinary mixture of hexane isomers on powder CAU-10(Al), an isostructural form of MIL-160(Al), at 423 K and total hydrocarbon pressure of 50 kPa. **Table S6** summarizes the experimental conditions applied and the equilibrium loadings.

**Figure S18** shows the multicomponent breakthrough curves for an equimolar septenary mixture of pentanes and hexanes isomers on powder MIL-160(Al) at 50.0 kPa. The effect of temperature is shown: a) 373 and b) 473 K. The experiment performed at 423 K is reported in the main text (Figure 5a). **Table S7** summarizes the experimental conditions applied, the equilibrium loadings, and selectivities.

**Figure S19** shows the multicomponent breakthrough curves for an equimolar quinary mixture of branched pentanes and hexane isomers on powder MIL-160(Al) at 50 kPa. The effect of temperature is shown: a) 373 and b) 473 K. The experiment performed at 423 K is reported in the main text (Figure 5b). The experimental conditions applied for these experiments are the same as those for the septenary mixtures shown in Table S7.

**Figure S20** shows the pure component adsorption equilibrium isotherms of pentane and hexane isomers on shaped MIL-160(Al) at 373, 423, 473 K and hydrocarbon pressures ranging from 2.5 to 50 kPa. **Table S8** summarizes the experimental conditions applied to measure the pure component breakthrough curves and the equilibrium loadings.

**Figure S21** shows the breakthrough curves for an equimolar binary mixture of hexane isomers on shaped MIL-160(Al) at 423 K and total hydrocarbon pressure of 50.0 kPa: a) 22DMB/23DMB, b) 23DMB/2MP, c) 3MP/2MP, and d) 2MP/nC6. **Table S9** summarizes the experimental conditions applied and the equilibrium loadings.

**Figure S22** shows the breakthrough curves for an equimolar ternary mixture of hexane isomers on shaped MIL-160(Al) at 423 K and total hydrocarbon pressure of 50.0 kPa: a) 22DMB/2MP/nC6 and b) 23DMB/2MP/nC6. The experimental conditions applied for these experiments are the same as those for the binary mixtures shown in Table S9.

**Figure S23** shows the multicomponent breakthrough curve for an equimolar quinary mixture of hexane isomers on shaped MIL-160(Al) at 50 kPa. The effect of temperature is shown: a) 423 K and b) 473 K. **Table S10** summarizes the experimental conditions applied, the equilibrium loadings, and selectivities.

**Figure S24** shows the multicomponent breakthrough curves for a septenary mixture of branched pentane and hexane isomers on shaped MIL-160(Al) at 423 K and 50.0 kPa using a concentration reported by Holcombe et al.,[6] which corresponds to the composition of the product stream from an isomerization reactor, as indicated in **Table S3**. The experimental conditions for this experiment and the respective equilibrium loadings are shown in **Table S11**.

**Figure S25** shows the multicomponent breakthrough curves for a quinary mixture of branched pentane and hexane isomers on shaped MIL-160(Al) at 423 K and 50.0 kPa, using a concentration reported by Holcombe et al.,[6] which corresponds to the composition of the product stream from an

isomerization reactor, considering the branched isomers as the only paraffins in the mixture as indicated in Table S3. The experimental conditions applied for this experiment are those given in Table S11.

**Figure S26** shows the multicomponent breakthrough curve for an equimolar septenary mixture of pentane and hexane isomers on a mixed bed (panel a) and layer bed (panel b) of shaped MIL-160(Al) (70 wt%)/ Zeolite 5A (30 wt%) at 423 K and 50 kPa. **Table S12** summarizes the experimental conditions applied. These experiments revealed that the mixed bed appears to be more effective in displacing the linear alkane nC5 wavefront from the middle of the HRON and LRON fractions. While nC5 was moved forward the mono-branched hexanes (2MP/3MP), only a "partial" displacement was seen in the layered bed, with the nC5 eluting practically together with these components. As a consequence, the 92 RON productivity obtained in the mixed-bed (1.14 mol $dm^{-3}$, highlighted in section 2.3 of the manuscript) was reduced by around 35 % (0.84 mol $dm^{-3}$) which is also lower than that achieved for the separation of hexane isomers only (1.02 mol $dm^{-3}$). Apart from this, the sorption dynamics of the other isomers remains practically the same.

**Figure S27** shows the multicomponent breakthrough curve for an equimolar septenary mixture of pentane and hexane isomers on a mixed bed of shaped MIL-160(Al) (70%)/ Zeolite 5A (30 wt%) at 473 K and 50 kPa. The experiment performed at 423 K is presented in the main text (Figure 6a). The experimental conditions applied for this experiment are those given in Table S12.

**Figure S28** shows the preliminary PSA tests for an equimolar septenary mixture of pentane and hexane isomers on a mixed bed of shaped MIL-160(Al) (70%) and binder-free beads Zeolite 5A (30 wt%) at 473 K and 50 kPa. The experiment performed at 423 K is presented in the main text (Figure 6b). **Table S13** summarizes the experimental conditions applied and the equilibrium loadings.

# 6. COMPUTATIONAL DETAILS

## 6.1 Configurational-bias Monte Carlo calculations

Configuration-bias Monte Carlo (CBMC) simulations were performed to calculate the adsorption isotherms of pure component and equimolar binary/quinary/septenary mixtures for pentane/hexanes isomers in MIL-160(Al) at 423 K. The crystal structure of MIL-160(Al) was taken from our previous study.[1] Since the guest-induced reorientation of the linkers has been shown to impact the adsorption uptakes in MOF frameworks with small to moderate pore size,[7] CBMC calculations were first performed to assess the impact of the rotational flexibility of the furan linkers in MIL-160(Al) framework on the pure component hexanes adsorption isotherms.

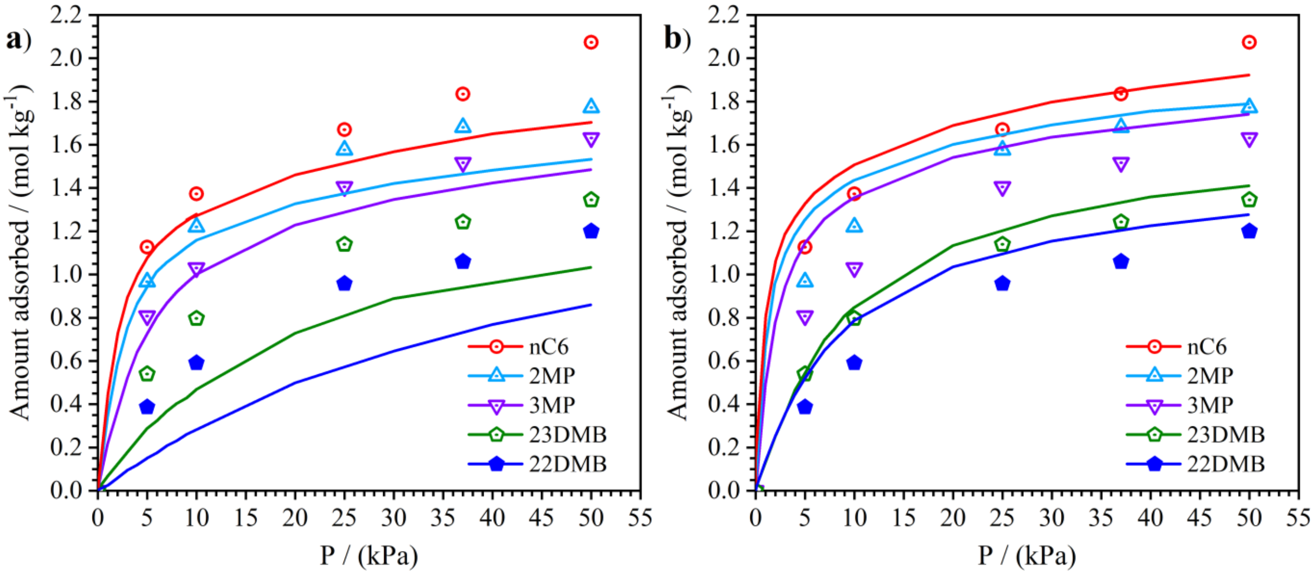


**Figure S30** reveals that a 5° rotation of the furan along the two carboxylate C atoms leads to an overall fair agreement between the experimental and CBMC simulated adsorption hierarchy for the hexane isomers. Notably, this relatively small tilting that accounts for a sub-angstrom level change in pore size (5.7 Å vs. 5.9 Å) yields the same adsorption sequence — *n*-C6 > 2MP > 3MP > 23DMB > 22DMB — observed in the pure component hexane isomers' adsorption measurements. The slight deviation between the experimental and simulated uptakes — particularly at higher pressure — can explain that a single configuration with identical tilting for all the linkers is likely unable to capture the whole adsorption regime. Importantly, the slopes of the simulated and experimental isotherms are in good agreement, which led us to believe that further calculations on this tilted furan linker configuration of MIL-160(Al) should provide reliable predictions on the multicomponent adsorption properties.

These CBMC simulations were performed with RASPA code.[8] We considered a simulation box made of 16 conventional unit cells (2 × 2 × 4) of the tilted MIL-160(Al) configuration maintaining atoms at their initial positions. The interactions between the guest molecules and the MOF structure were

described by a combination of site-to-site Lennard-Jones (LJ) potentials. Furthermore, van der Waals interactions were truncated at a cutoff distance of 12 Å. The Lennard-Jones cross-interaction parameters were calculated by means of the Lorentz-Berthelot mixing rules. Typically, $5\times10^5$ and $1\times10^5$ cycles (a cycle consists further 20 Monte Carlo moves) have been used for equilibration and production runs. These guest alkanes were modeled using the Transferable Potentials for Phase Equilibria United Atom (TraPPE-UA) models, i.e., molecules are represented by pseudo atoms: $CH_3$, $CH_2$, CH, and C with implicit hydrogen atoms.[9,10] The intramolecular C-C bond lengths of the molecules are set at 1.540 Å, and the bond angles differ between 109.5° and 114°, depending on the bond type. The intermolecular LJ potential parameters are listed in **Table S14**. Atoms of the MOF framework were described by single LJ sites with parameters taken from the universal force field (UFF)[11] except for the Al and H atoms for which the LJ interactions were neglected as previously reported.[1] Henry's constant ($K_H$) for each of the pentane/hexanes was calculated using the revised Widom's test particle insertion method.

### 6.2 DFT host-guest interaction energy calculations

The host-guest interaction energies for each C5 and C6 isomer were computed using dispersion corrected DFT and the periodic boundary conditions, and applying the same settings described above. In doing so, a single molecule of the isomers was inserted into the MIL-160 1x1x2 super cell model via NVT Monte Carlo simulations and further fully DFT-geometry optimized. The adsorption binding energies for each C5/C6-isomer were calculated using the following. formula: $E_{\mathrm{Int}} = E_{\mathrm{MOF+guest\text{-}molecule}} - (E_{\mathrm{MOF}} + E_{\mathrm{guest\text{-}molecule}})$, where $E_{\mathrm{MOF+guest\text{-}molecule}}$ corresponds to the energy of the DFT-optimized geometry for the different C5/C6 isomers loaded MIL-160 configuration, $E_{\mathrm{MOF}}$ and $E_{\mathrm{guest\text{-}molecule}}$ correspond to the energies of the empty MIL-160 and an individual C5 and C6 isomer, respectively.

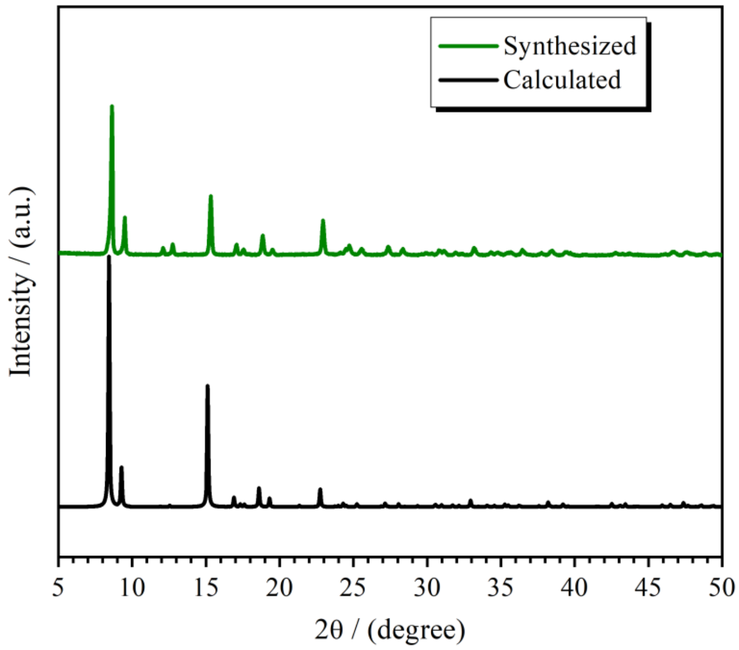


**Figure S1**. PXRD patterns of MIL-160(Al).

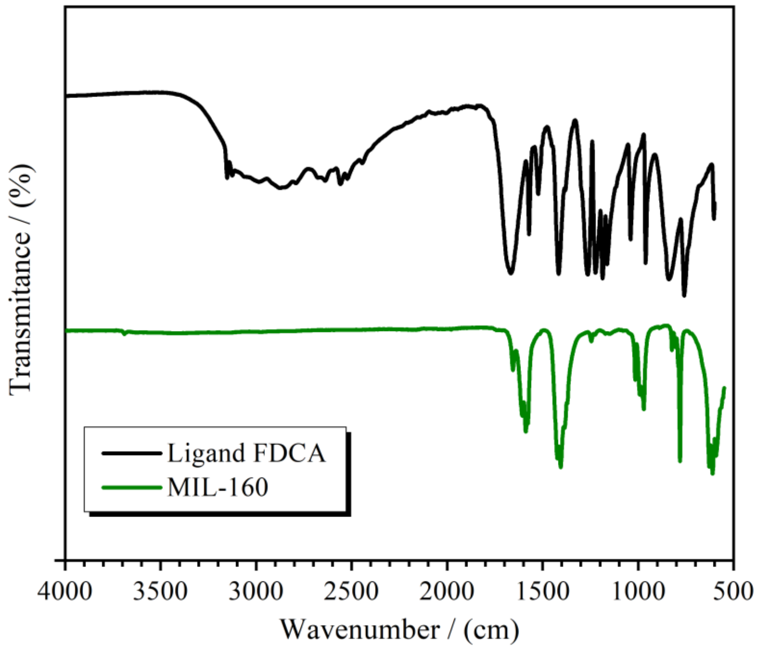


**Figure S2**. FTIR spectra of MIL-160(Al) and organic linker FDCA.

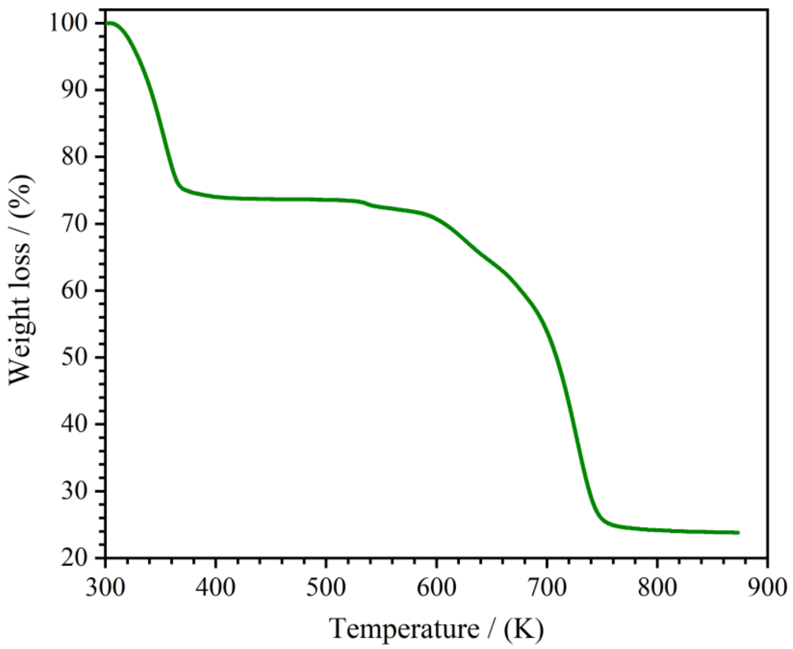


**Figure S3**. TGA analysis of MIL-160(Al).

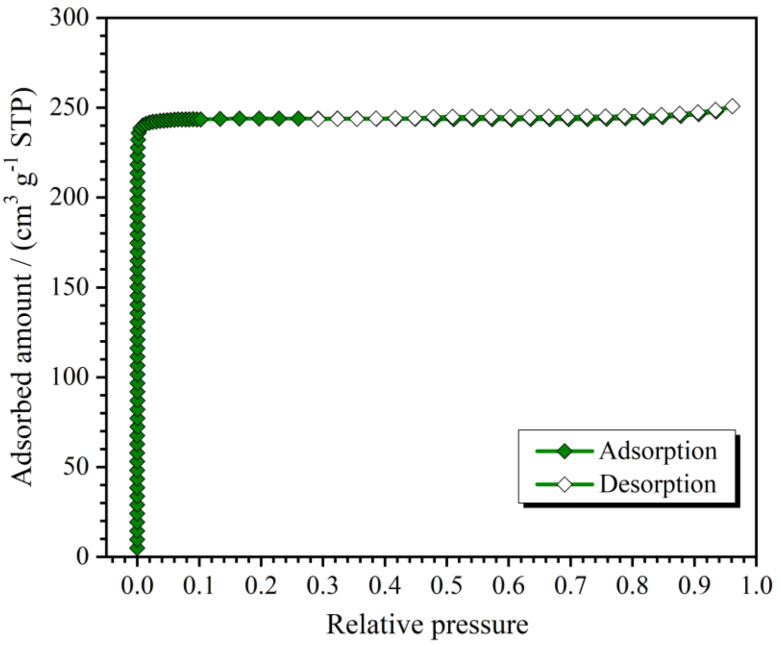


**Figure S4**. Nitrogen adsorption and desorption isotherms at 77 K on MIL-160(Al).

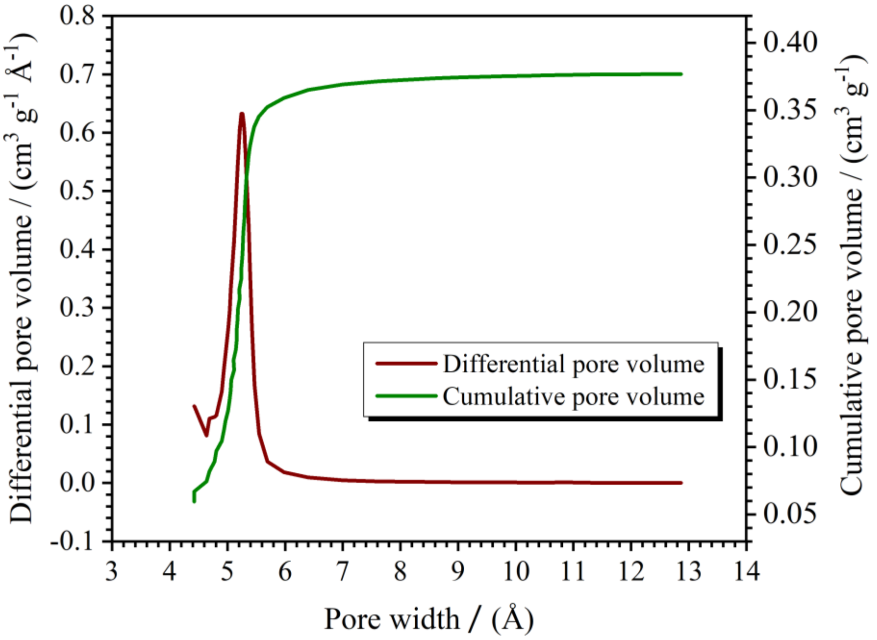


**Figure S5**. Pore size distribution and cumulative pore volume of MIL-160(Al).

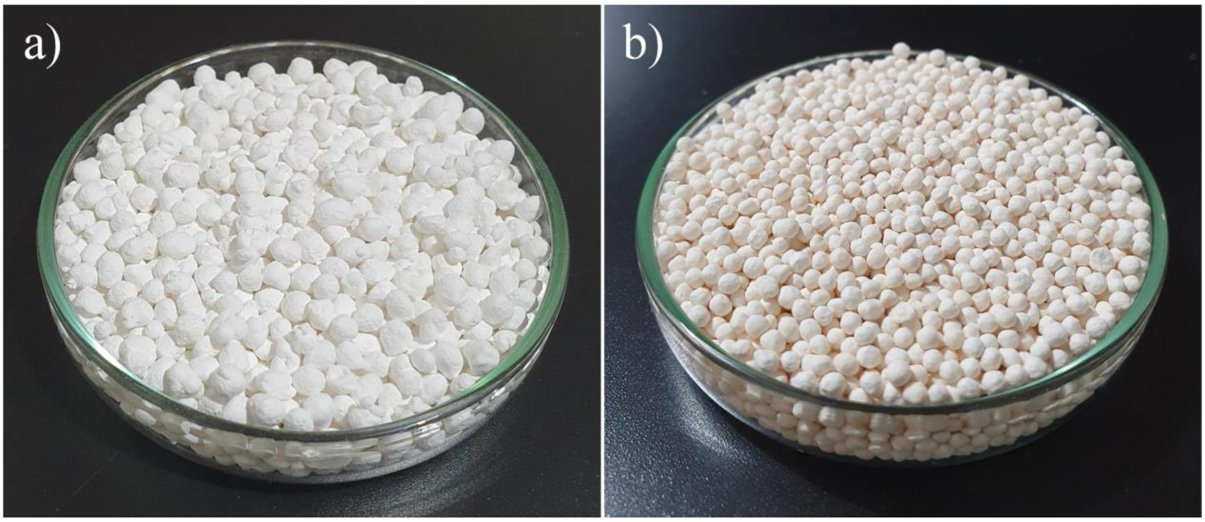


**Figure S6**. Adsorbent materials in the shaped form: a) MIL-160(Al) beads (2.0 to 3.35 mm). b) Zeolite 5A binder-free beads (1.2 to 2.0 mm)

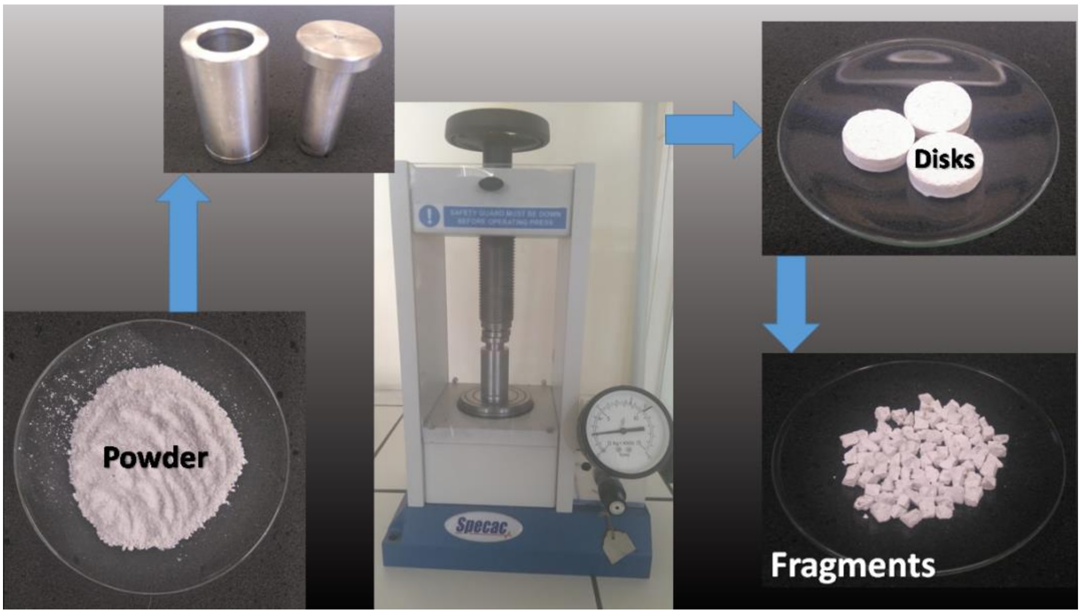


**Figure S7**. Experimental procedure for agglomeration of powdered samples of MIL-160(Al).

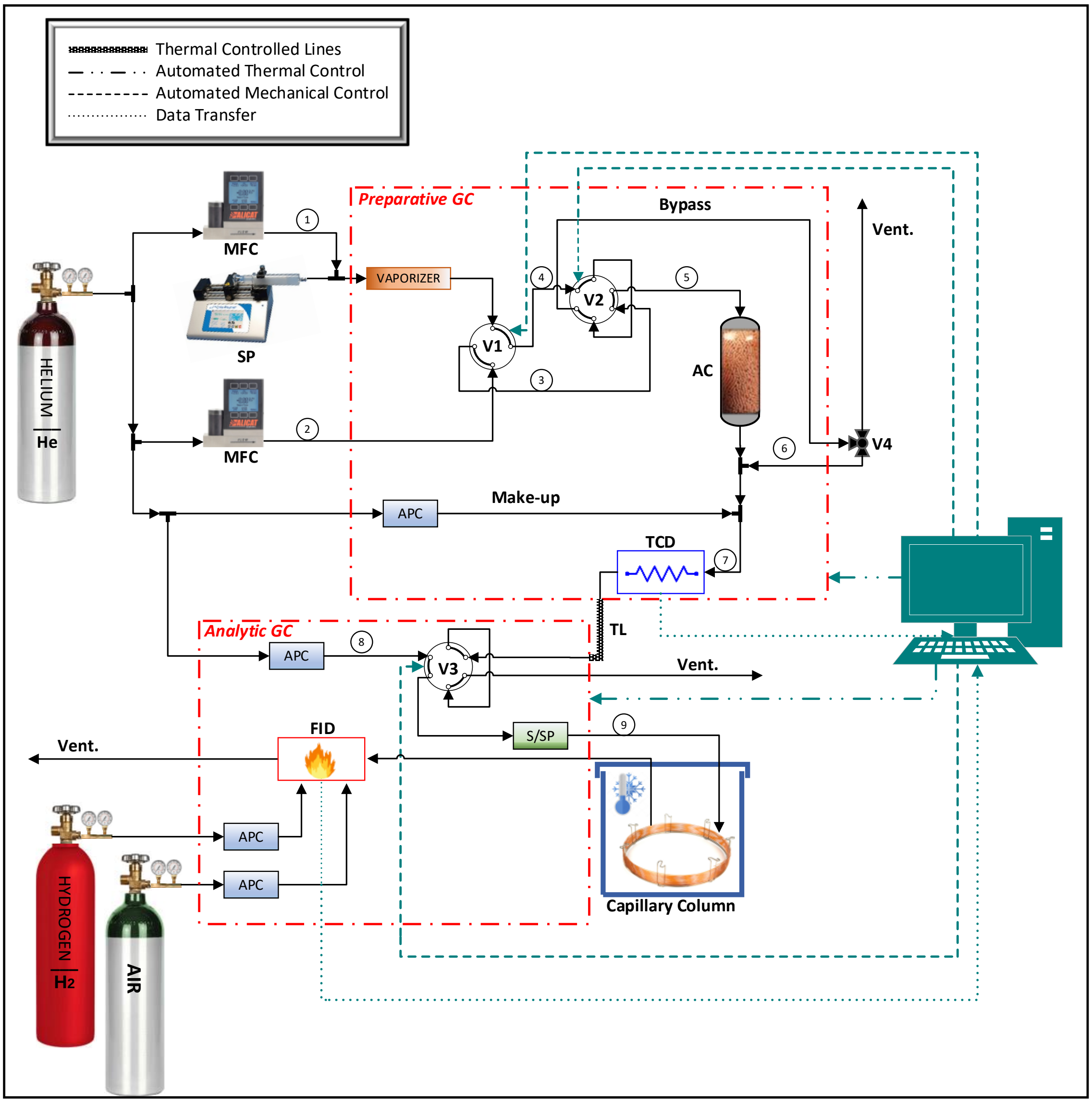


**Figure S8**. Schematic diagram of the experimental apparatus used to measure single and multicomponent breakthrough curves. (AC) adsorption column; (APC) advanced pneumatic control; (FID) flame ionization detector; (MFC) mass flow controller; (S/SP) split/splitless-injector; (SP) syringe pump; (TCD) thermal conductivity detector; (TL) transfer line; (V1) 4-way valve; (V2) and (V3) 6-way valve; (V4) 3-way valve.

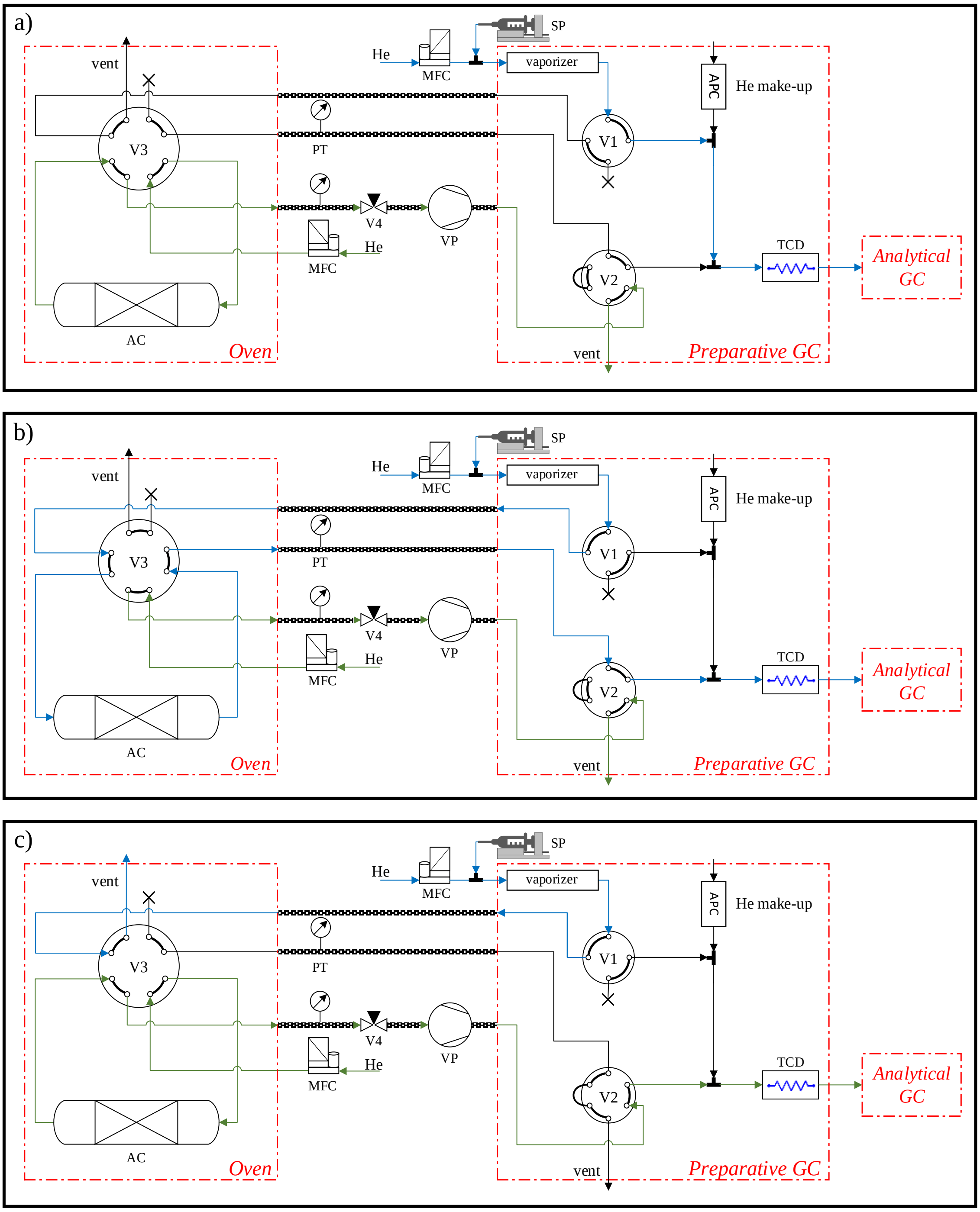


**Figure S9**. Schematic diagram of the experimental apparatus used to perform the continuous cyclic experiments: a) Gas preparation section for checking the paraffins vaporization; b) Pressurization with feed and adsorption; and c) Vacuum countercurrent depressurization with inert He purge desorption. Blue and green lines represent the paraffins (adsorption) and He purge (desorption) flow paths inside the system, respectively.

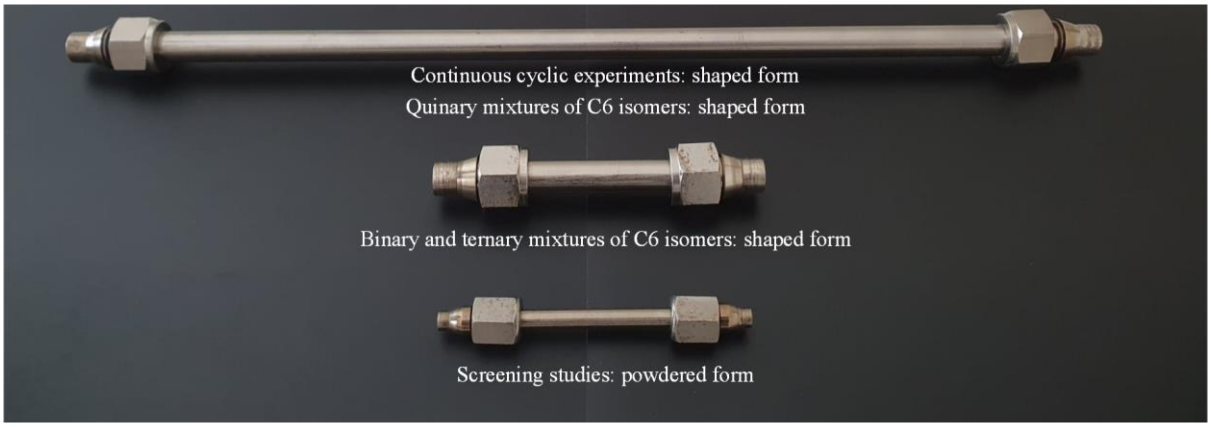


**Figure S10**. Adsorption columns used in the sorption experiments.

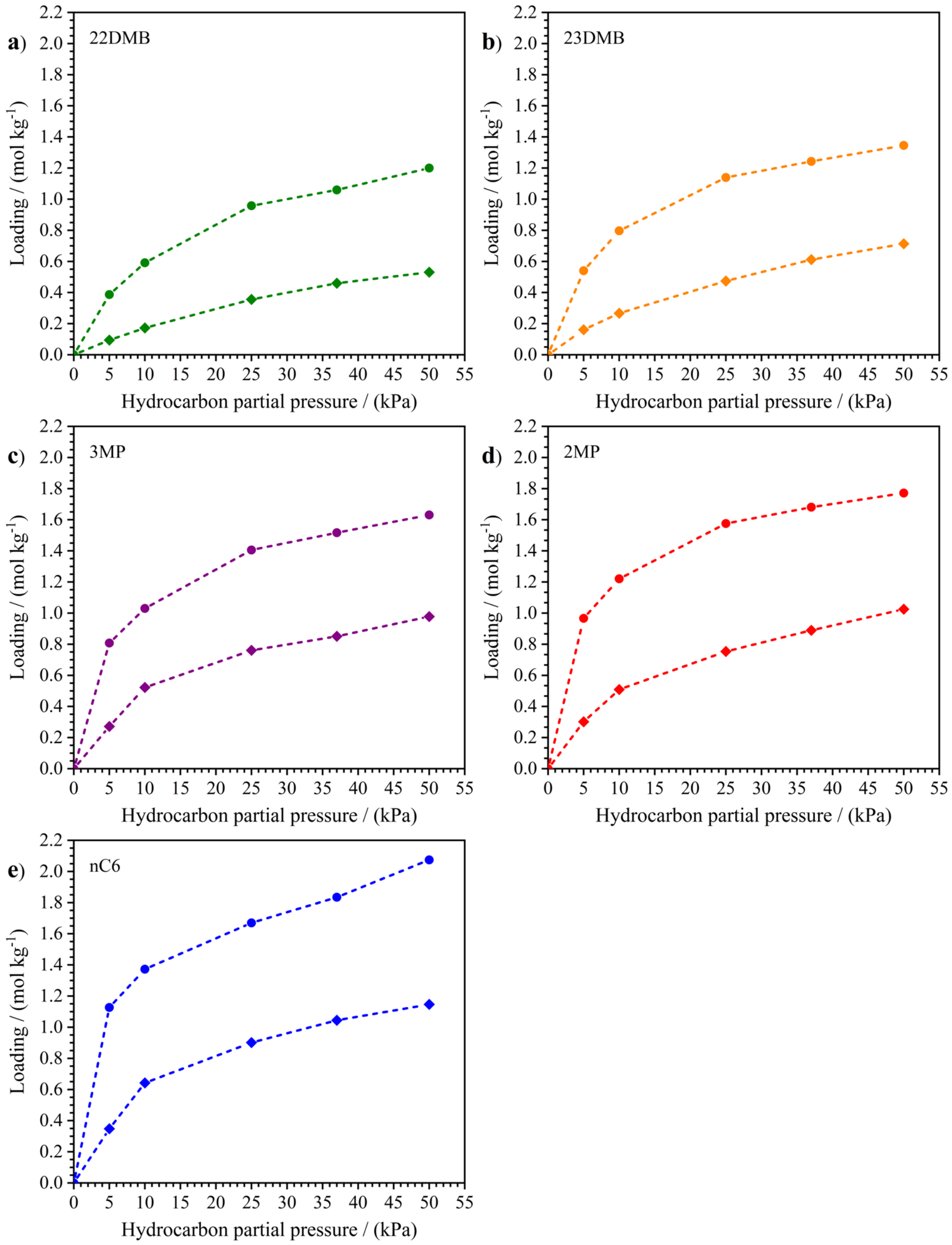


**Figure S11**. Experimental pure component adsorption equilibrium isotherms of hexane isomers on powder MIL-160(Al). a) 22DMB, b) 23DMB, c) 3MP, d) 2MP, and e) nC6 at 423K (circles) and 473 K (diamonds)

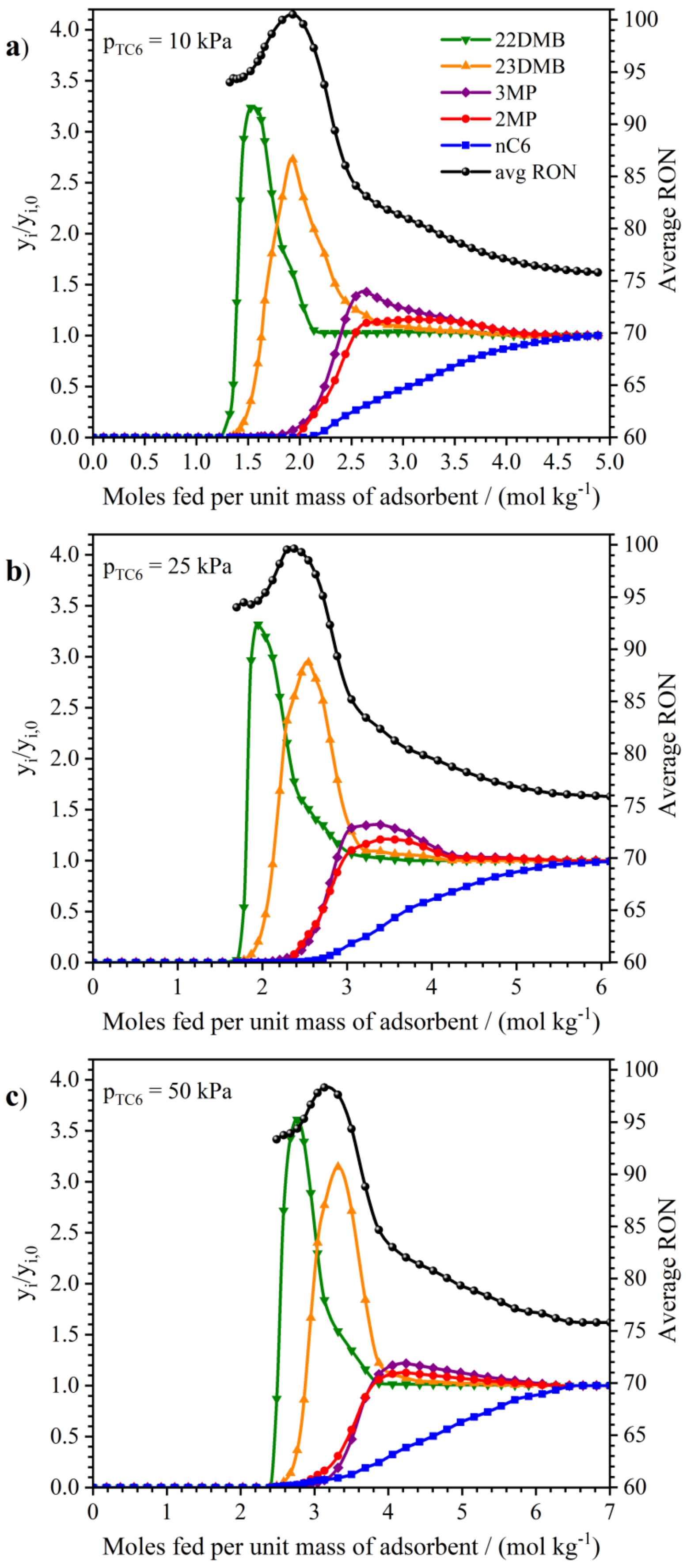


**Figure S12**. Experimental multicomponent breakthrough curves for an equimolar quinary mixture of hexane isomers on powder MIL-160 at 373 K and a) 10 kPa, b) 25.0 kPa, and c) 50kPa.

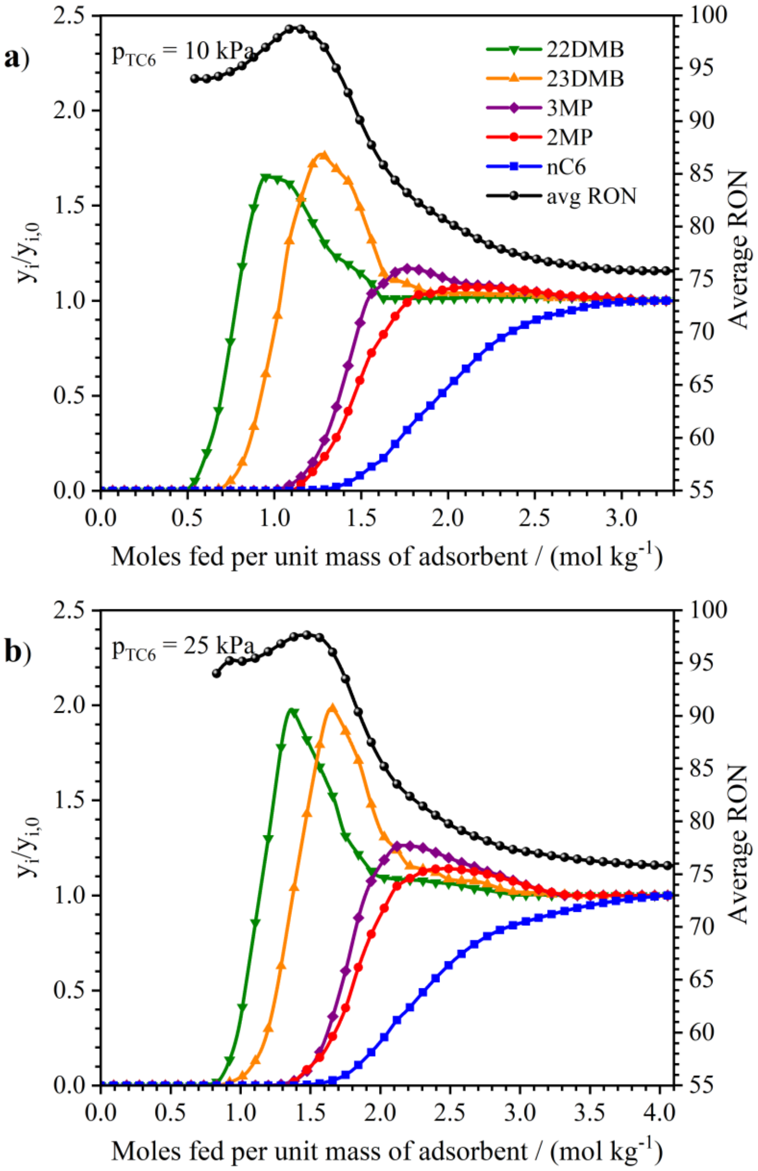


**Figure S13**. Experimental multicomponent breakthrough curves for an equimolar quinary mixture of hexane isomers on powder MIL-160 at 423 K and a) 10 kPa and b) 25 kPa.

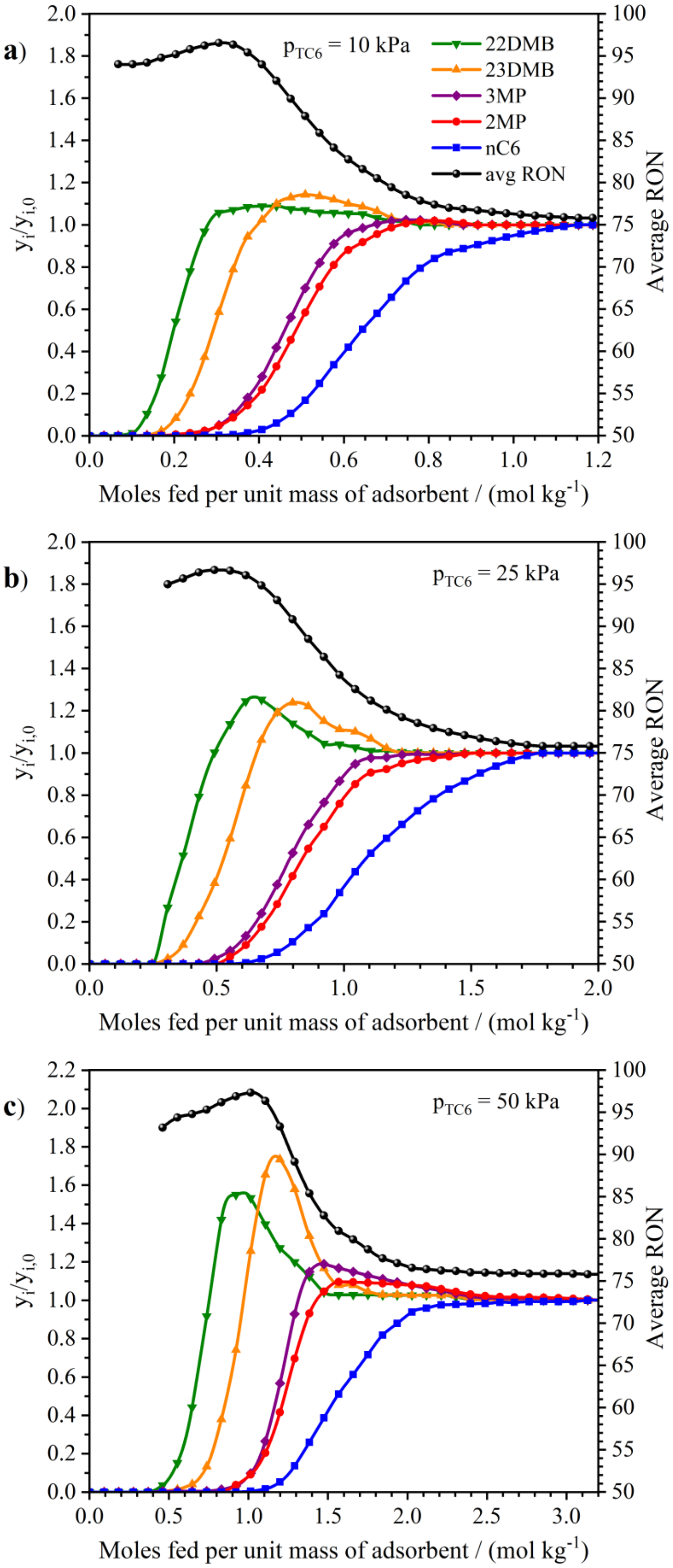


**Figure S14**. Experimental multicomponent breakthrough curves for an equimolar quinary mixture of hexane isomers on powder MIL-160 at 473 K and a) 10 kPa, b) 25.0 kPa, and c) 50kPa.

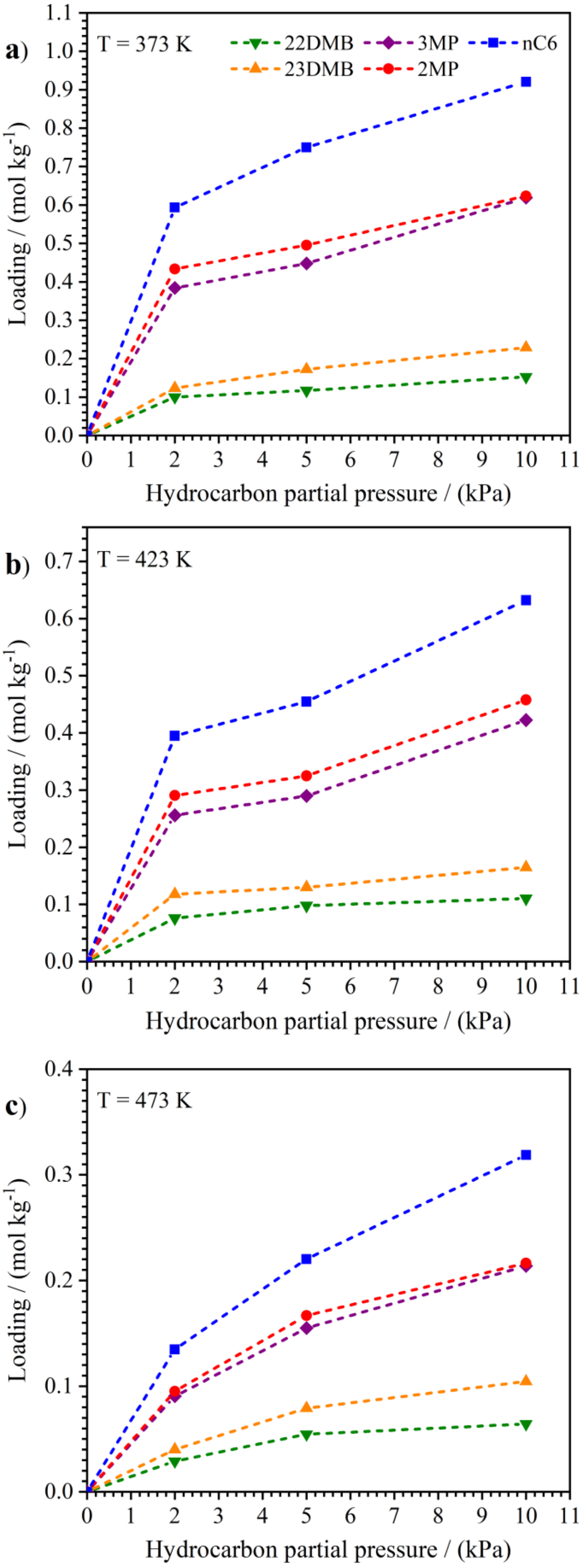


**Figure S15**. Experimental quinary adsorption equilibrium isotherms for an equimolar mixture of hexane isomers on powder MIL-160 at (a) 373 K, (b) 423 K, and (c) 473 K.

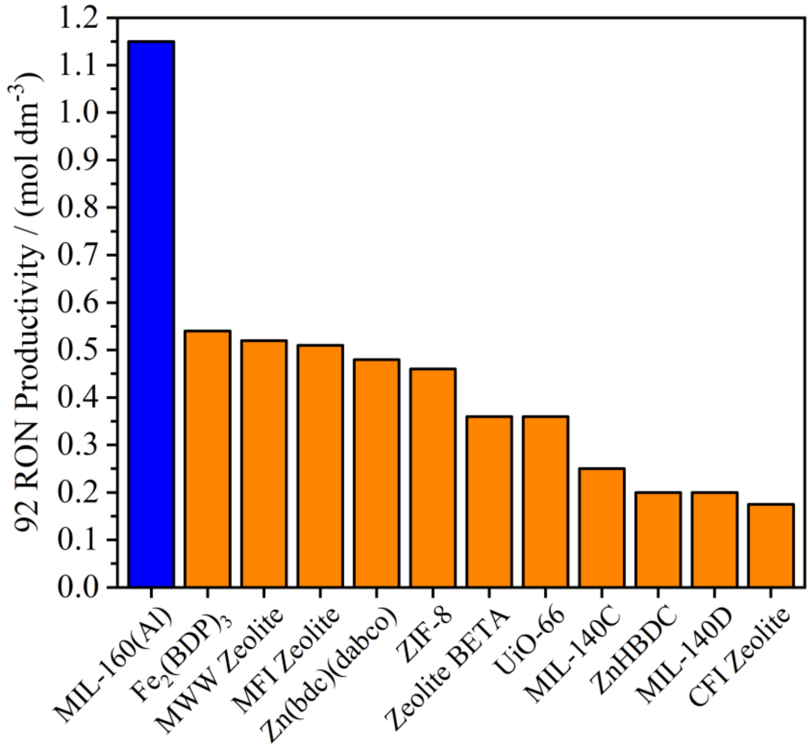


**Figure S16**. Comparison of the 92 RON productivities for separating equimolar quinary mixtures of hexane isomers (nC6/2MP/3MP/23DMB/22DMB) using a variety of materials under similar working conditions. The blue column represents the experimental data obtained on MIL-160(Al) at a total hydrocarbon pressure of 50 kPa ($p_{n\text{-}C6} = p_{2MP} = p_{3MP} = p_{23DMB} = p_{22DMB} = 10$ kPa) and temperature of 423 K, while all other columns in orange represents the data reported by Herm et al.,[5] which are based on CBMC simulations performed at a total hydrocarbon pressure of 100 kPa ($p_{n\text{-}C6} = p_{2MP} = p_{3MP} = p_{23DMB} = p_{22DMB} = 20$ kPa) and temperature of 433 K.

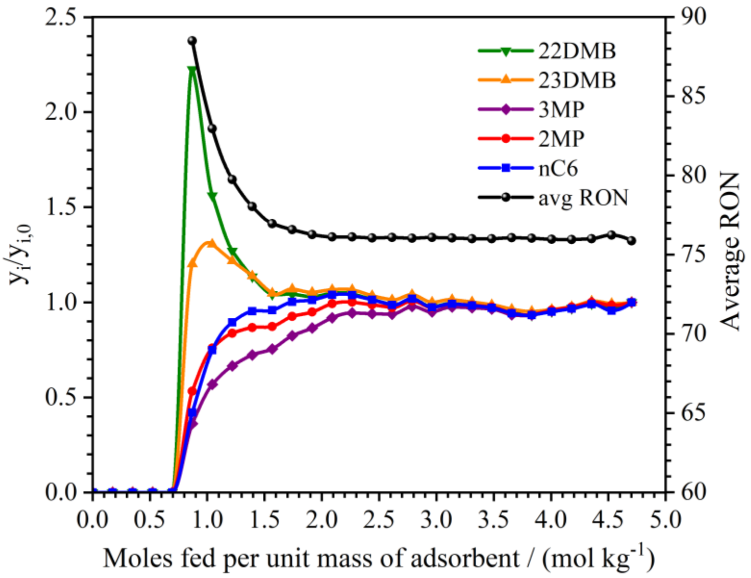


**Figure S17**. Experimental multicomponent breakthrough curves for an equimolar quinary mixture of hexane isomers on powder CAU-10 at 423 K and 50kPa.

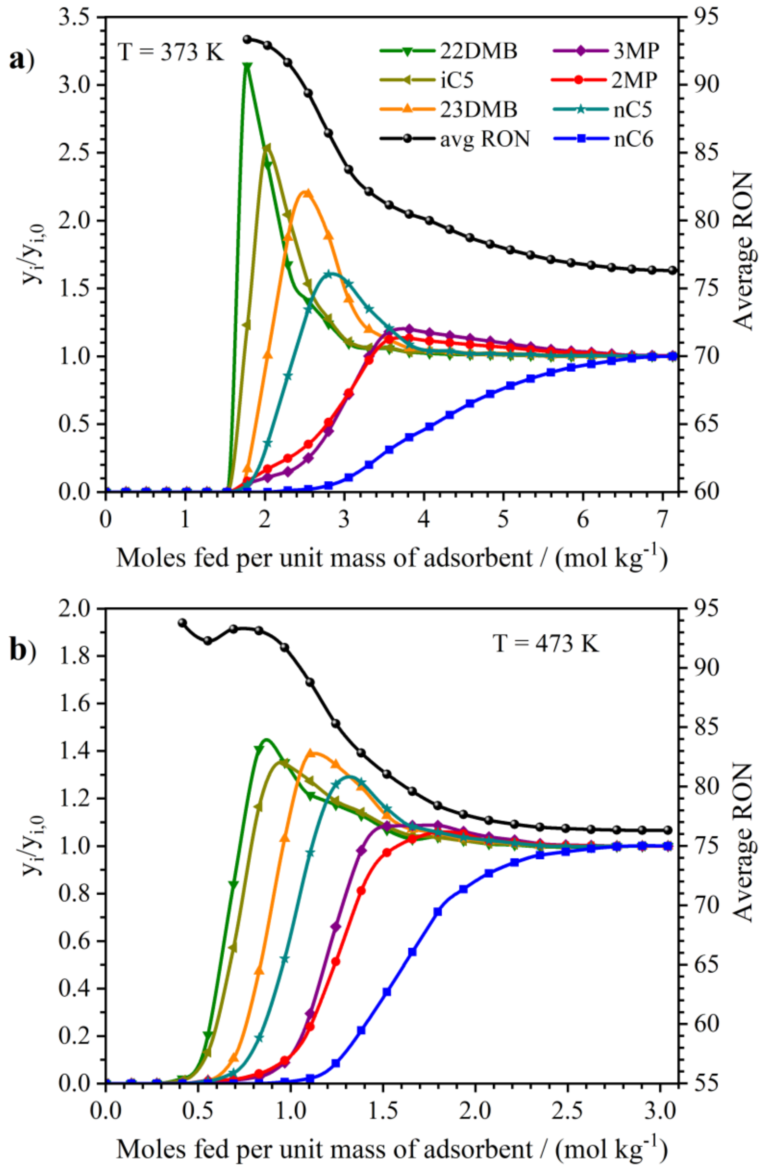


**Figure S18**. Experimental multicomponent breakthrough curves for an equimolar septenary mixture of pentane and hexane isomers on powder MIL-160 at 50 kPa and a) 373 K and b) 473 K.

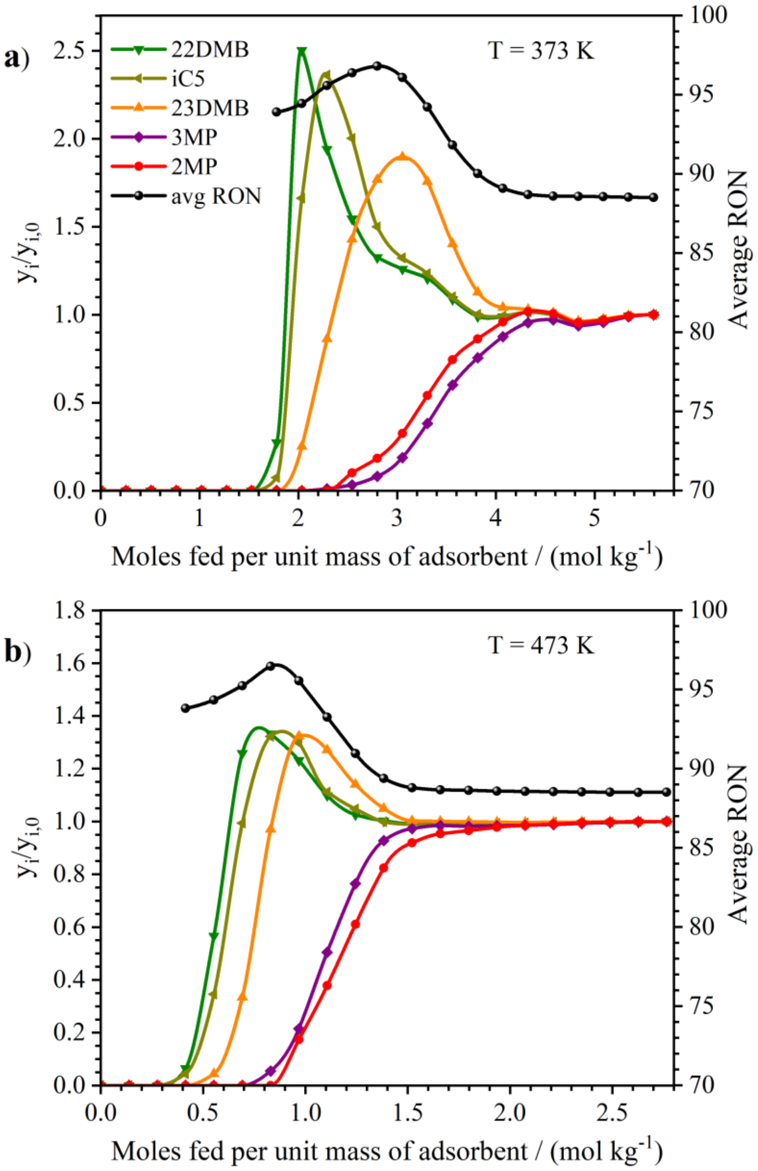


**Figure S19**. Experimental multicomponent breakthrough curves for an equimolar quinary mixture of branched pentane and hexane isomers on powder MIL-160 at 50 kPa and a) 373 K and b) 473 K.

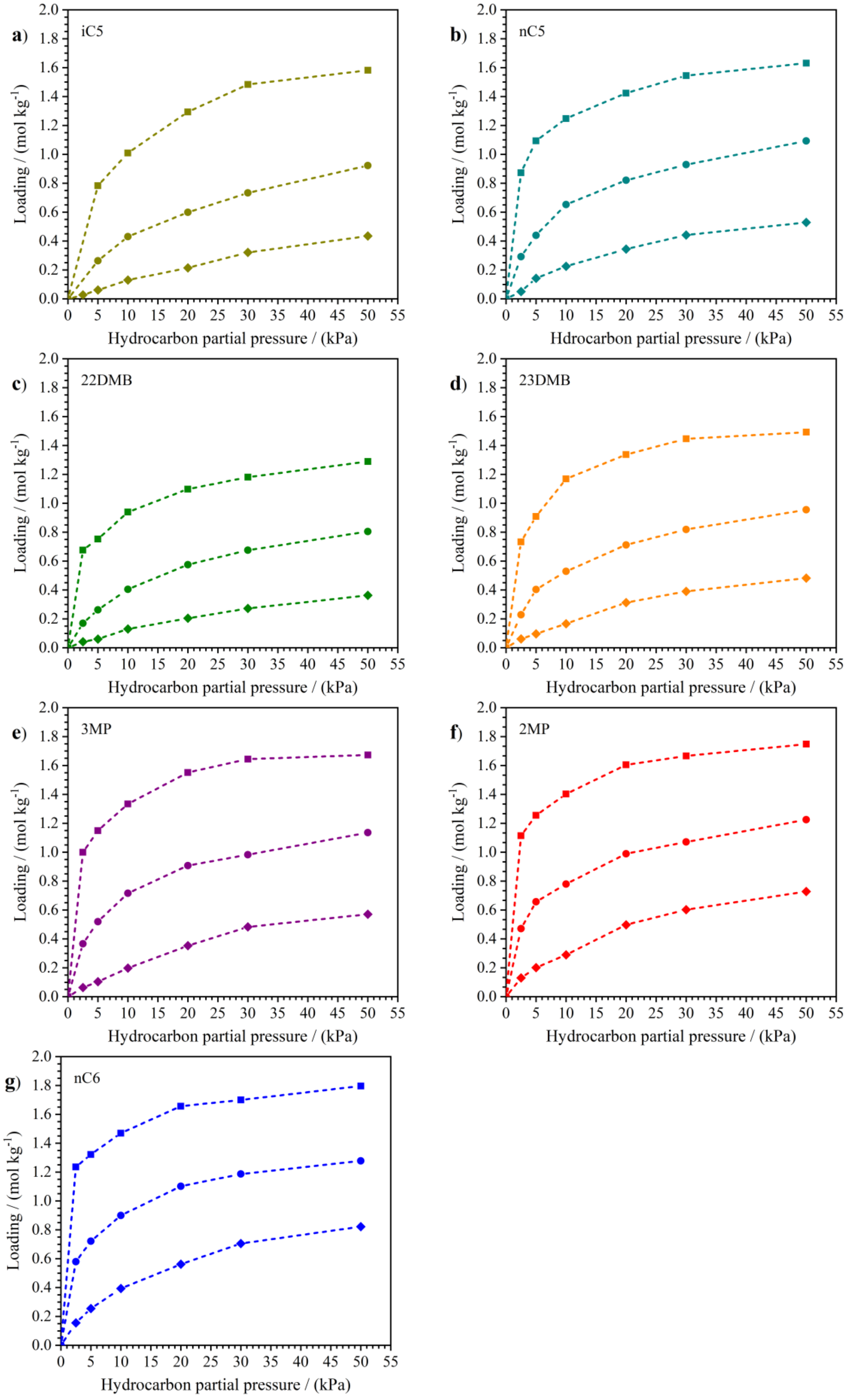


**Figure S20**. Experimental pure component adsorption equilibrium isotherms of pentane and hexane isomers on shaped MIL-160(Al). a) iC5, b) nC5, c) 22DMB, d) 23DMB, e) 3MP, f) 3MP, and g) nC6 at 373 K (squares), 423K (circles), and 473 K (diamonds).

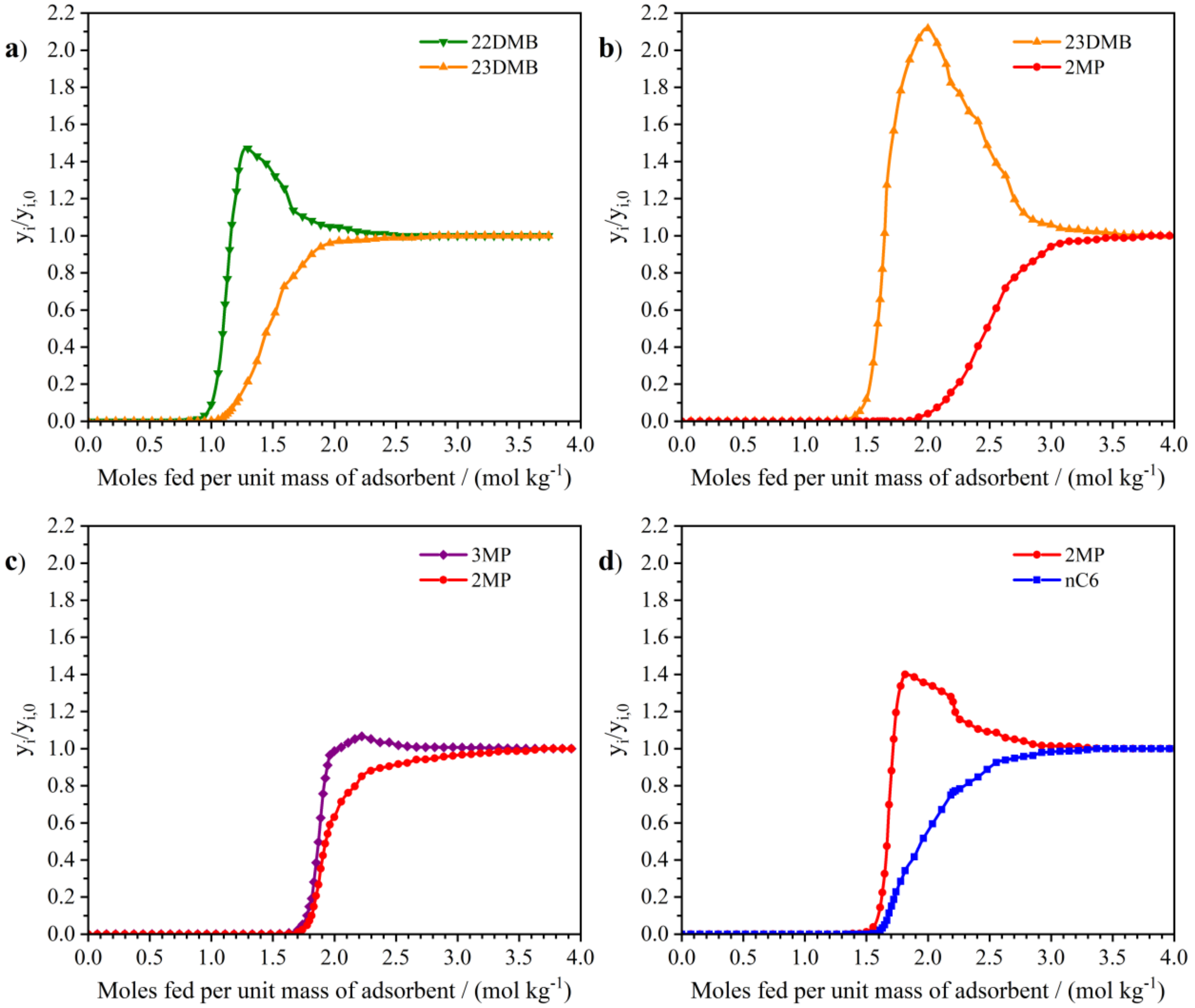


**Figure S21**. Experimental multicomponent breakthrough curves for an equimolar binary mixture of hexane isomers on shaped MIL-160 at 423 K and 50 kPa. a) 22DMB/23DMB, b) 23DMB/2MP, c) 3MP/2MP, and d) 2MP/nC6.

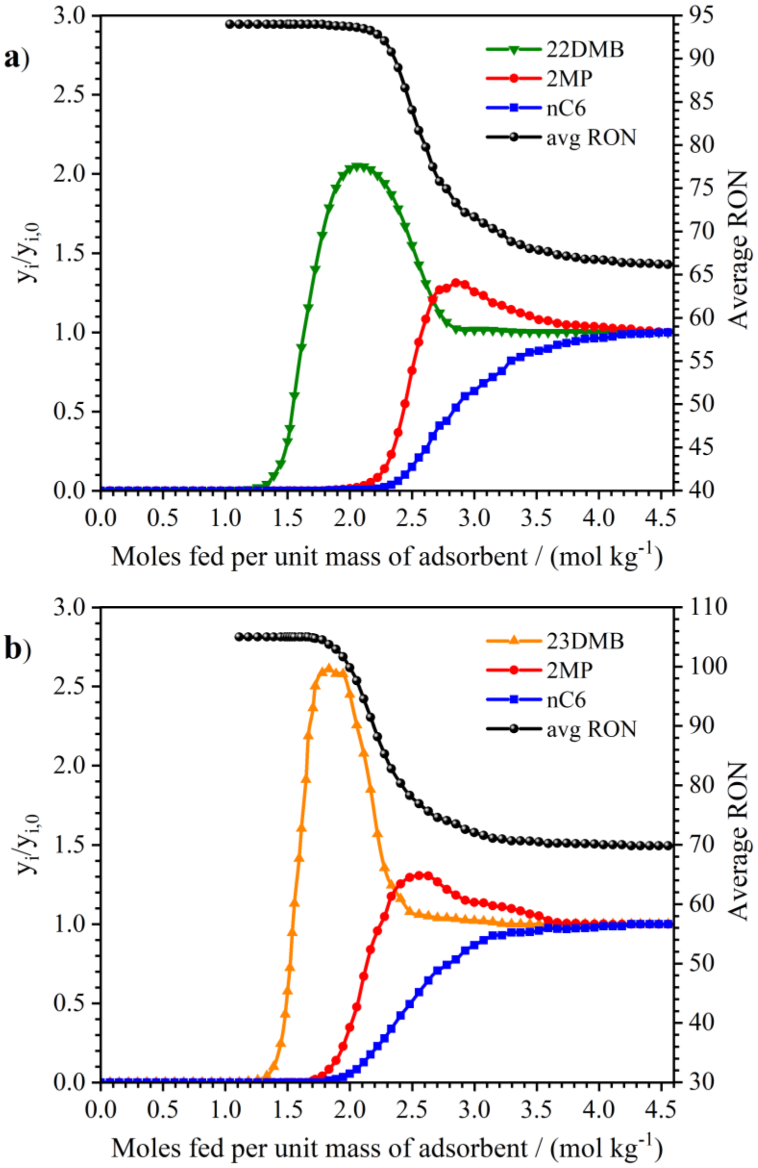


**Figure S22**. Experimental multicomponent breakthrough curves for an equimolar ternary mixture of hexane isomers on shaped MIL-160 at 423 K and 50 kPa. a) 22DMB/2MP/nC6 and b) 23DMB/2MP/nC6

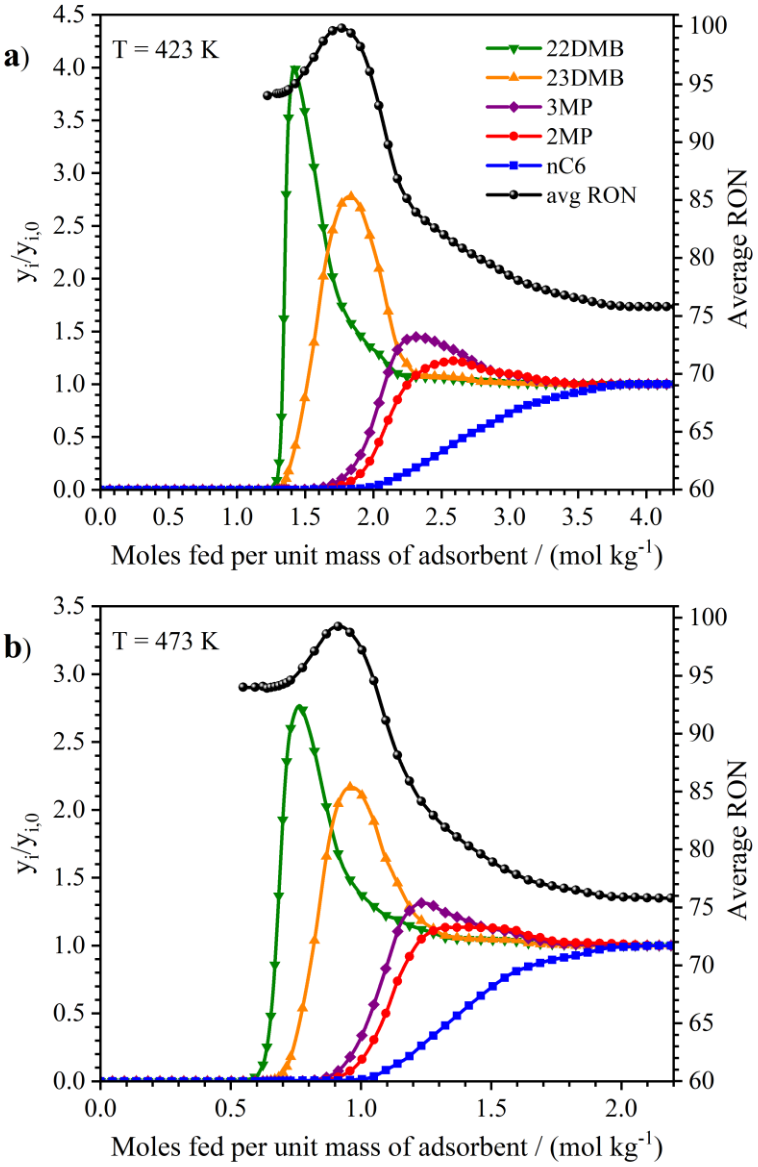


**Figure S23**. Experimental multicomponent breakthrough curves for an equimolar quinary mixture of hexane isomers on shaped MIL-160 at 50 kPa and a) 423 K and b) 473 K.

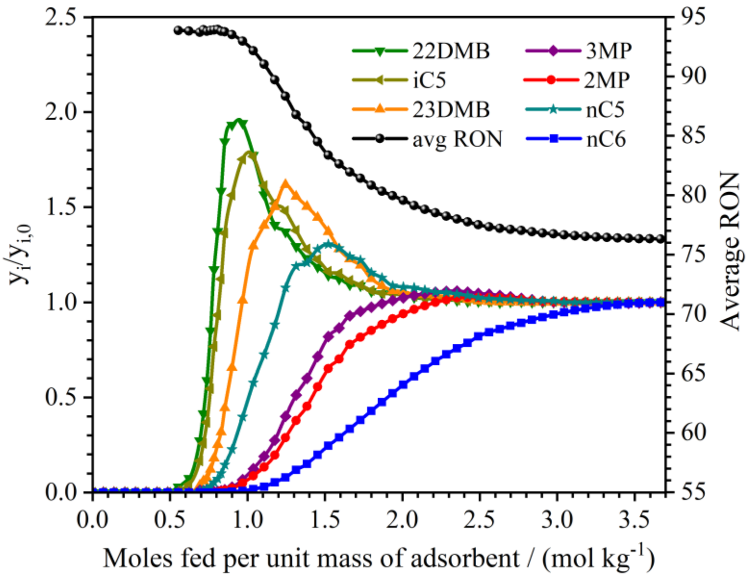


**Figure S24**. Experimental multicomponent breakthrough curves for a septenary mixture of pentane and hexane isomers on shaped MIL-160 at 423 K and 50 kPa. The concentration used is equal to one reported by Holcombe et al.,[6] which corresponds to the composition of the product stream from an isomerization reactor.

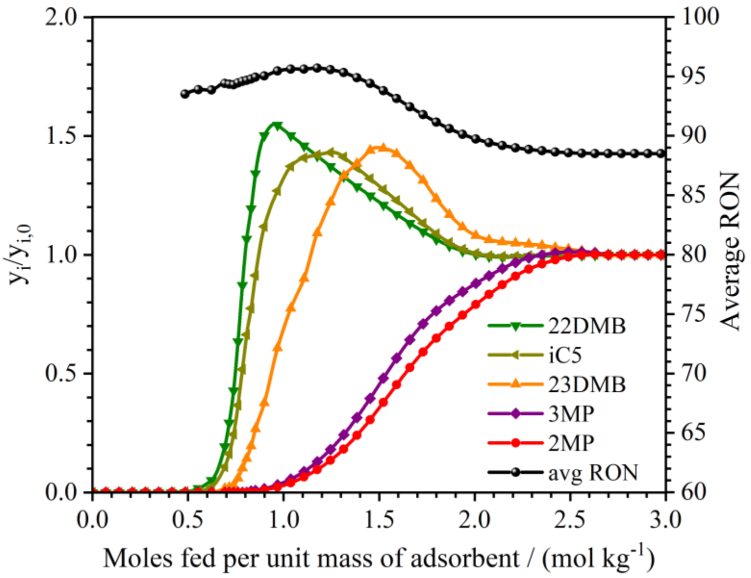


**Figure S25**. Experimental multicomponent breakthrough curves for a quinary mixture of pentane and hexane isomers on shaped MIL-160 at 423 K and 50 kPa. The concentration used is equal to one reported by Holcombe et al.,[6] which corresponds to the composition of the product stream from an isomerization reactor, considering the branched isomers as the only paraffins in the mixture.

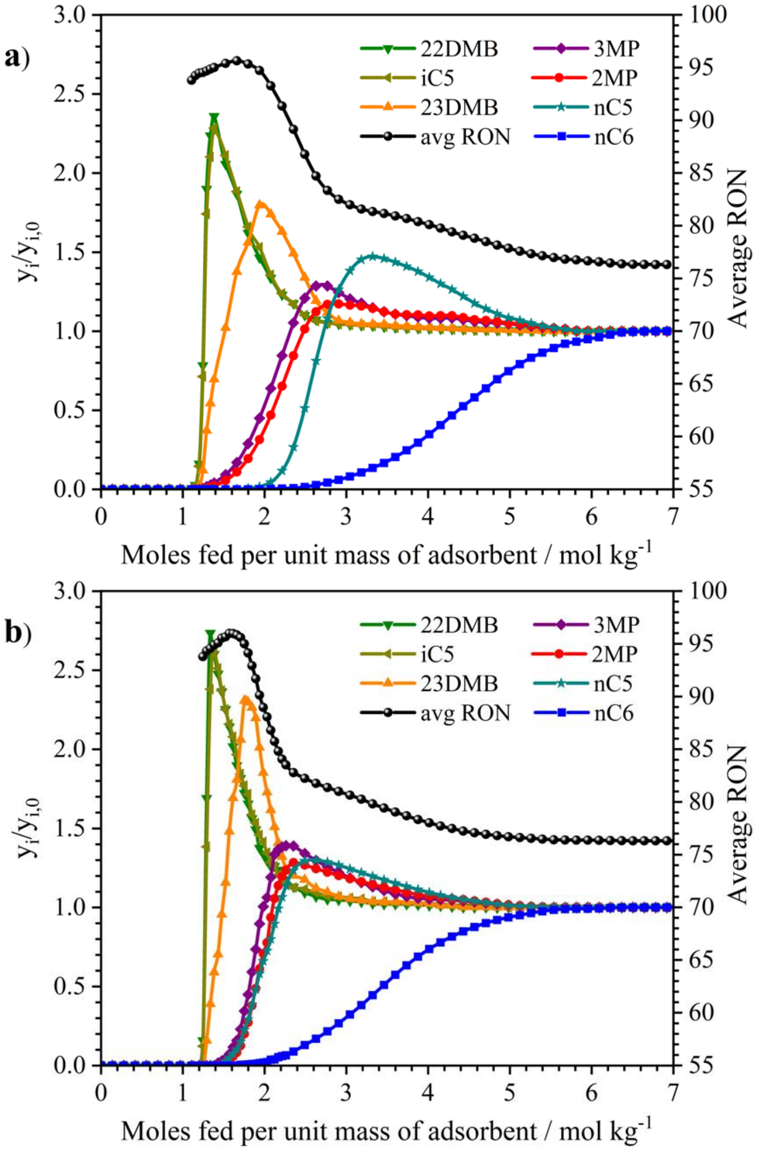


**Figure S26**. Experimental multicomponent breakthrough curves for an equimolar septenary mixture of pentane and hexane isomers on a bed of shaped MOF MIL-160(Al) (70 wt%) and binder-free beads Zeolite 5A (30 wt%) at 423 K and 50 kPa. a) Mixed bed and b) Layered bed (mixture passing through the zeolite 5A packing first).

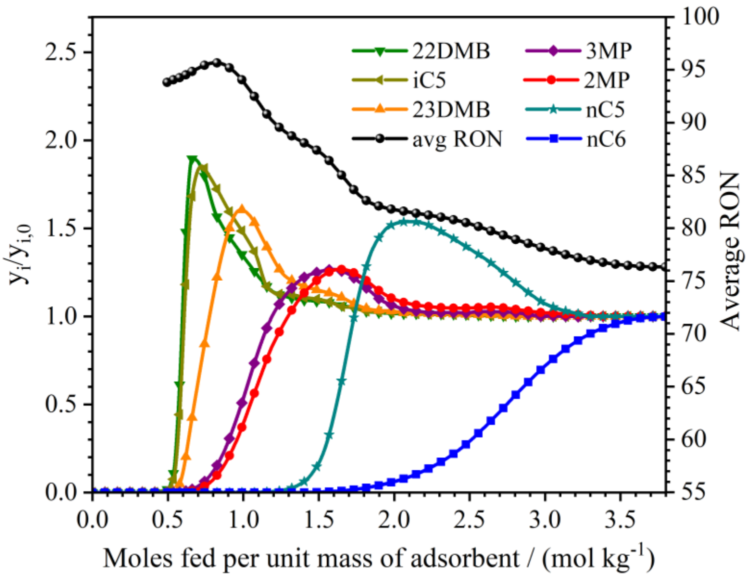


**Figure S27**. Experimental multicomponent breakthrough curves for an equimolar septenary mixture of hexane isomers on a mixed bed of shaped MOF MIL-160(Al) (70 wt%) and binder-free beads Zeolite 5A (30 wt%) at 473 K and 50 kPa.

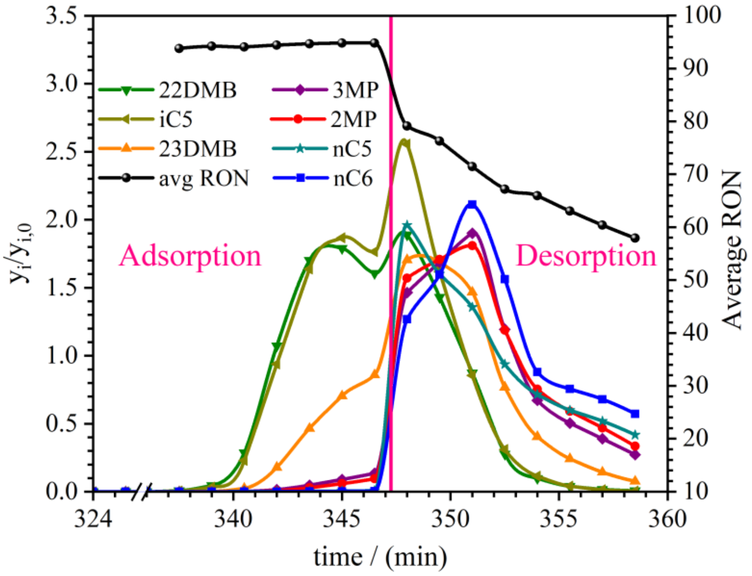


**Figure S28**. Experimental preliminary PSA test for an equimolar septenary mixture of pentane and hexane isomers on a mixed bed of shaped MOF MIL-160(Al) (70%) and binder-free beads Zeolite 5A (30 wt%) at 473 K and 50.0 kPa. Steady-state effluent concentration.

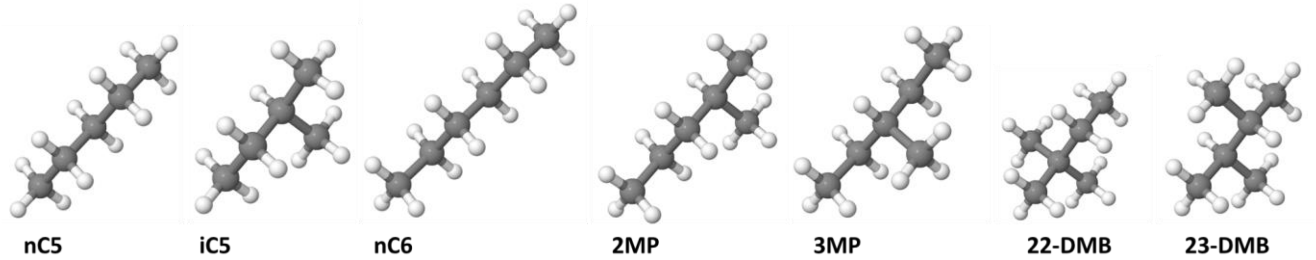


**Figure S29**. Molecular representation of the pentane/hexane isomers. Implicit hydrogen atoms are lumped onto the neighboring carbons and described with pseudo atoms $CH_3$, $CH_2$, CH, and C according to TraPPE-UA potentials.

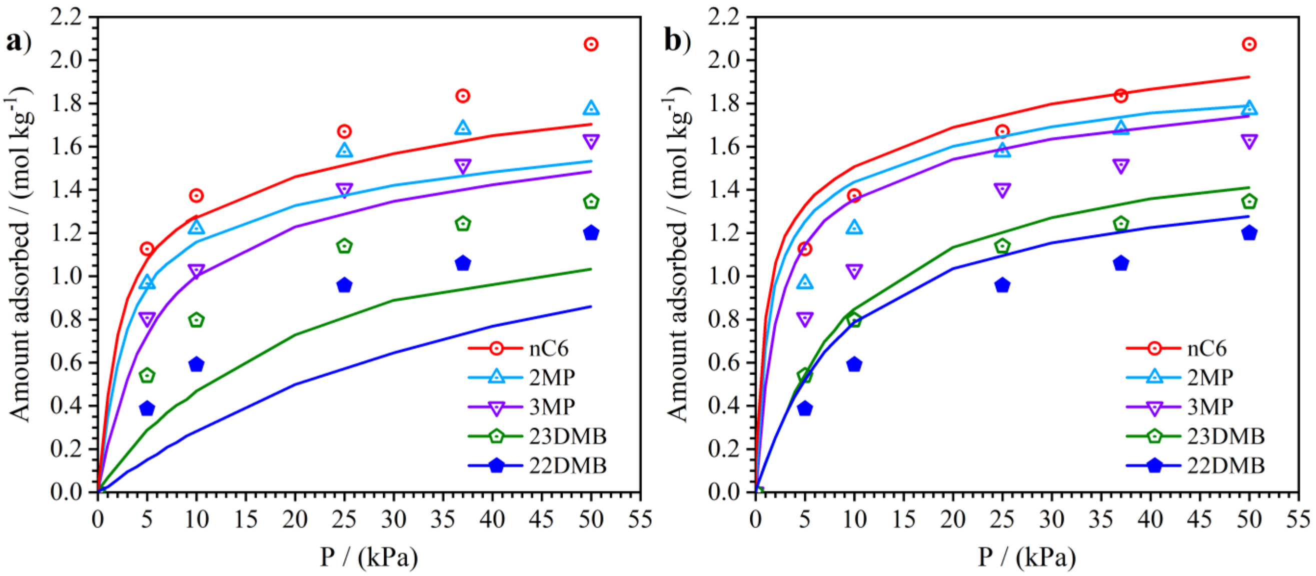


**Figure S30**. Experimental and simulated single component adsorption isotherms of hexane isomers on MIL-160(Al) for the (a) pristine and (b) and 5° linker rotation obtained at $T$ = 423 K. Markers represent the experimental data, and the continuous lines represent the CBMC simulations.

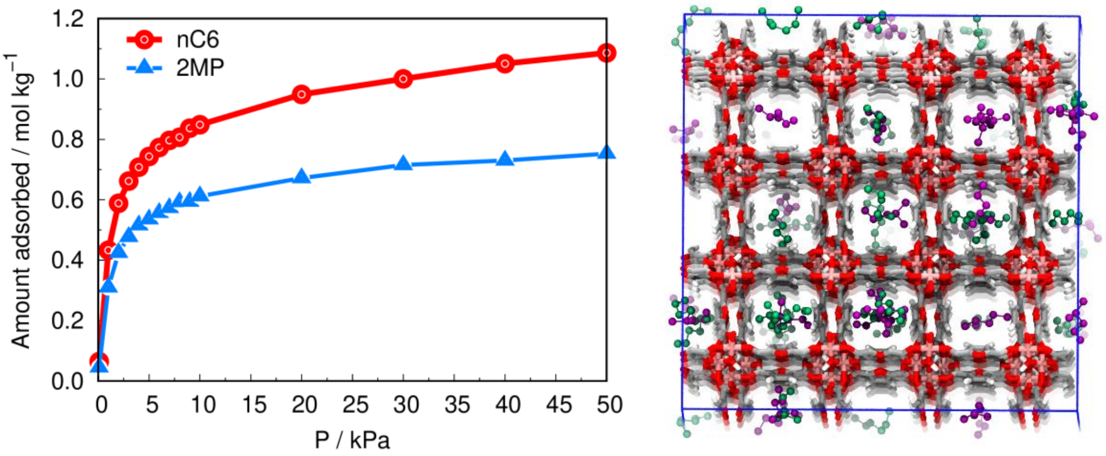


**Figure S31**. CBMC simulated co-adsorption isotherms of equimolar *n*-C6/2MP binary mixture for the MIL-160(Al) with 5° tilted linkers at $T$ = 423 K (left panel) and a snapshot of the adsorbed *n*-C6 (green spheres) and 2MP (purple spheres) molecules at $P$ = 10 kPa within the channel of the MOF (right panel).

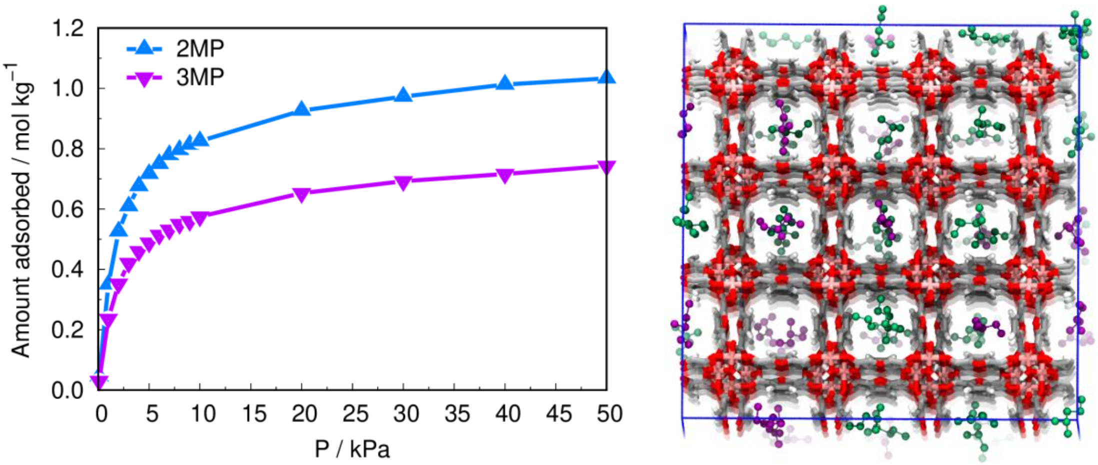


**Figure S32**. CBMC simulated co-adsorption isotherms of equimolar 2MP/3MP binary mixture for the MIL-160(Al) with 5° tilted linkers at *T* = 423 K (left panel) and a snapshot of the adsorbed 2MP (green spheres) and 3MP (purple spheres) molecules at *P* = 10 kPa within the channel of the MOF (right panel).

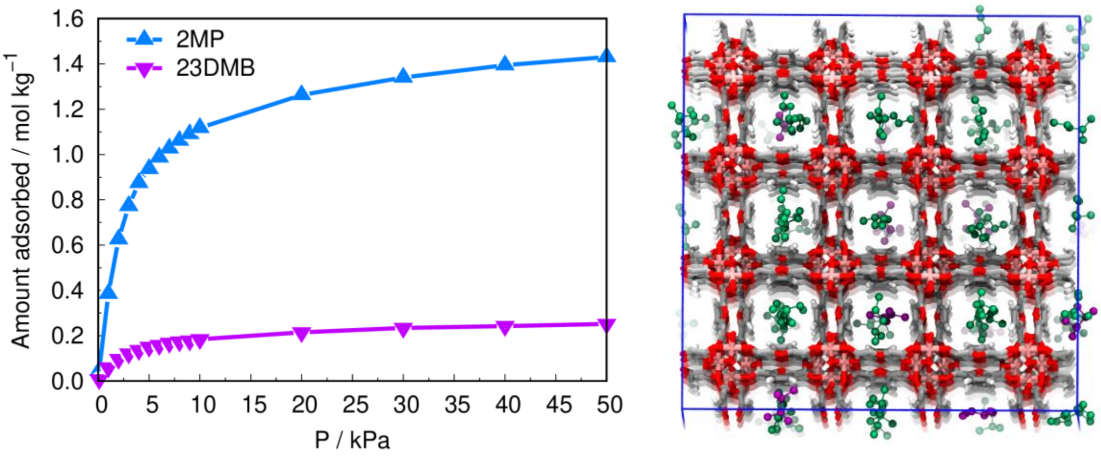


**Figure S33**. CBMC simulated co-adsorption isotherms of equimolar 2MP/23DMB binary mixture for the MIL-160(Al) with 5° tilted linkers at *T* = 423 K (left panel) and a snapshot of the adsorbed 2MP (green spheres) and 23DMB (purple spheres) molecules at *P* = 10 kPa within the channel of the MOF (right panel).

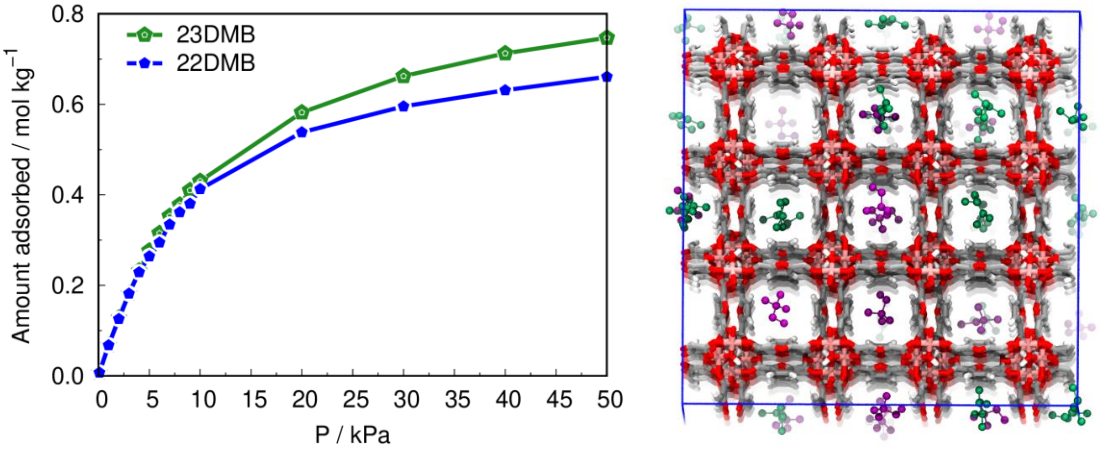


**Figure S34**. CBMC simulated co-adsorption isotherms of equimolar 22DMB/23DMB binary mixture for the MIL-160(Al) with 5° tilted linkers at $T$ = 423 K (left panel) and a snapshot of the adsorbed 22DMB (green spheres) and 23DMB (purple spheres) molecules at $P$ = 10 kPa within the channel of the MOF (right panel).

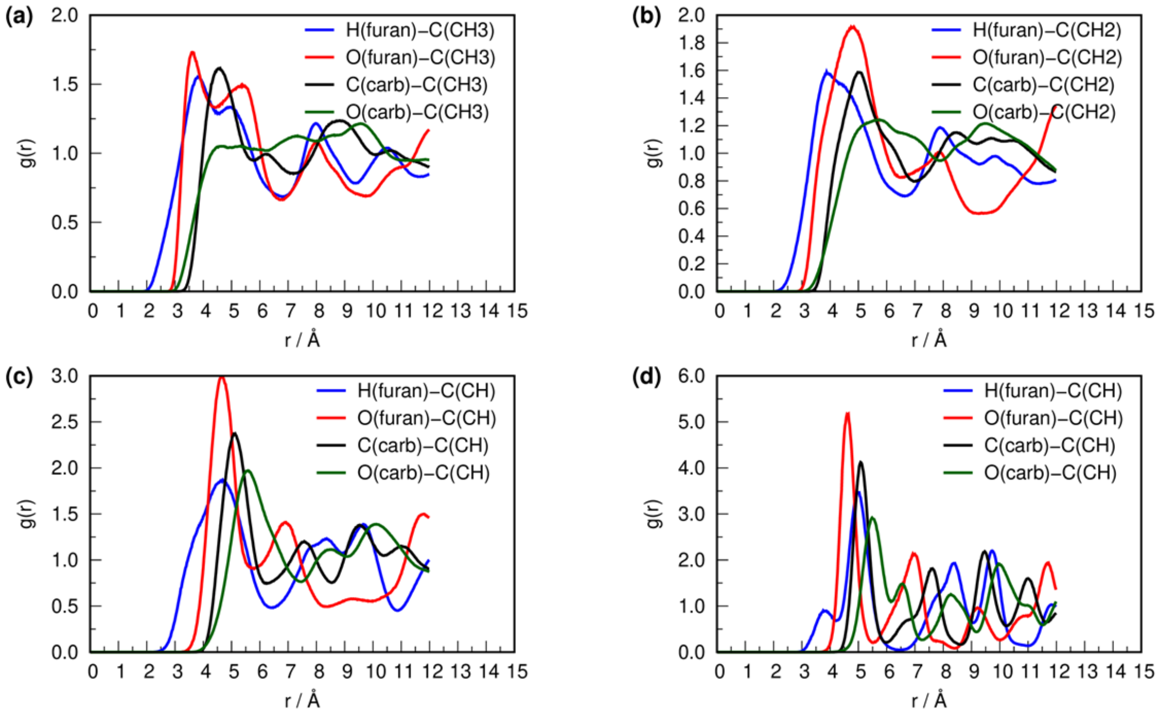


**Figure S35**. Intermolecular radial pair distribution functions of the (a) $CH_3$(UA), (b) $CH_2$(UA), (c) CH(UA), and (d) C of the hexane isomers with respect to MIL-160(Al) framework atoms in the vicinity of the pore channel calculated for an equimolar quinary mixture at $T$ = 423 K and $P$ = 10 kPa.

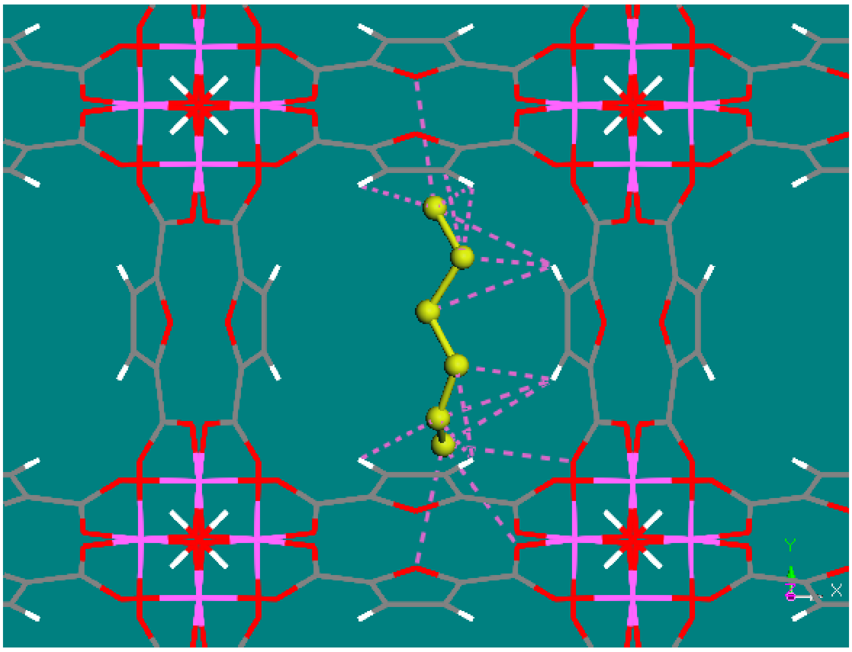

**Figure S36**. Illustration of a typical spatial arrangement and van der Waals close contacts (<3.5 Å) of an *n*-C6 molecule with the MIL-160(Al) pore walls taken from a representative adsorption snapshot derived from CBMC simulation performed for an equimolar quinary mixture of all hexane isomers at *T* = 423 K and *P* = 20kPa.

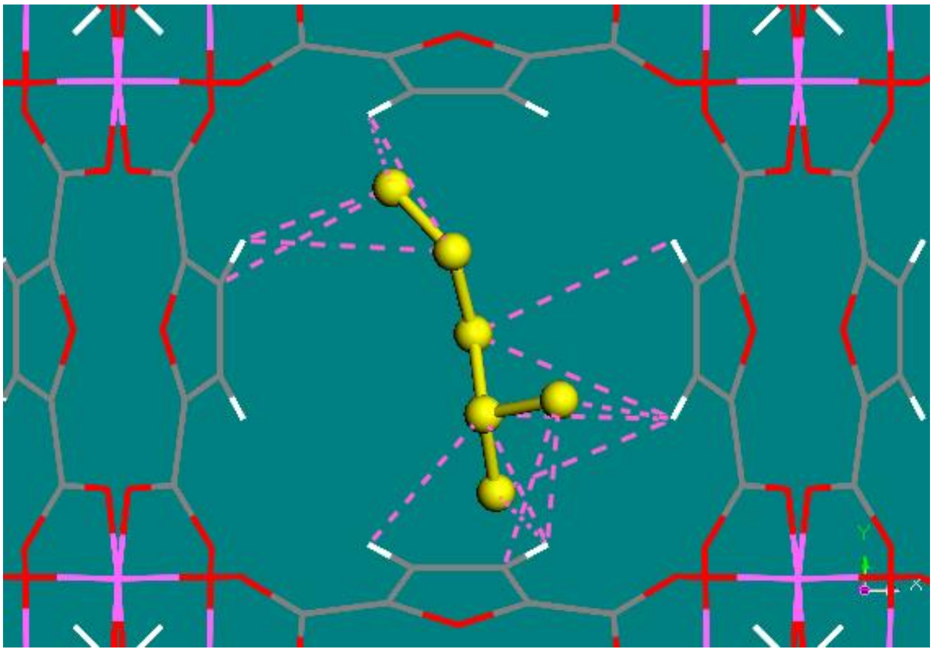

**Figure S37**. Illustration of a typical spatial arrangement and van der Waals close contacts (<3.5 Å) of a 2MP molecule with the MIL-160(Al) pore walls taken from a representative adsorption snapshot derived from CBMC simulation performed for an equimolar quinary mixture of all hexane isomers at $T$ = 423 K and $P$ = 20 kPa.

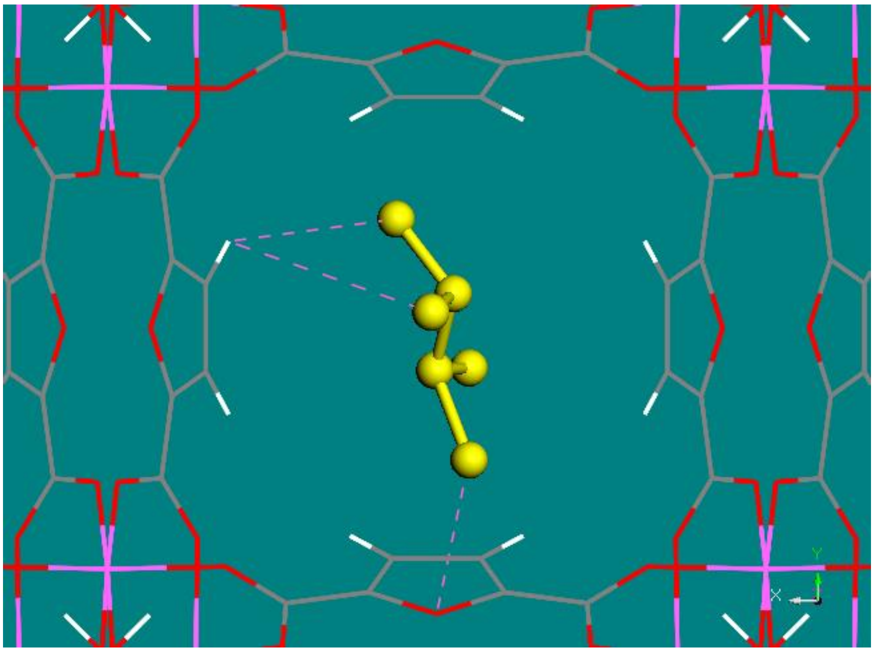

**Figure S38**. Illustration of a typical spatial arrangement and van der Waals close contacts (<3.5 Å) of a 23DMB molecule with the MIL-160(Al) pore walls taken from a representative adsorption snapshot derived from CBMC simulation performed for an equimolar quinary mixture of all hexane isomers at *T* = 423 K and *P* = 20 kPa.

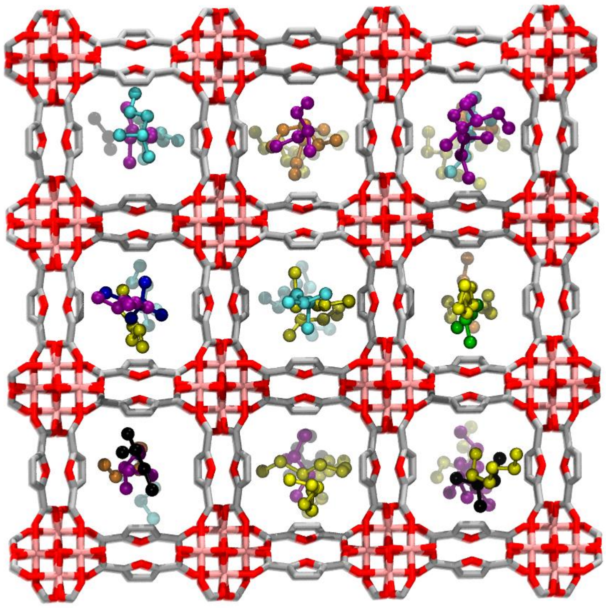

**Figure S39**. Illustration of CBMC simulated pore filling and spatial arrangement of all pentane and hexane isomers in the MOF channel obtained for an equimolar septenary mixture of C5/6 isomers at $T$ = 423 K and $P$ = 20 kPa. Color codes: *n*-C5 (orange spheres), iC5 (black spheres), *n*-C6 (yellow spheres), 2MP (cyan spheres), 3MP (purple spheres), 23DMB (green spheres) and 22DMB (blue spheres), MOF framework atoms: Al (pink), Carbon (grey) and Oxygen (red), hydrogen atoms are omitted for clarity.

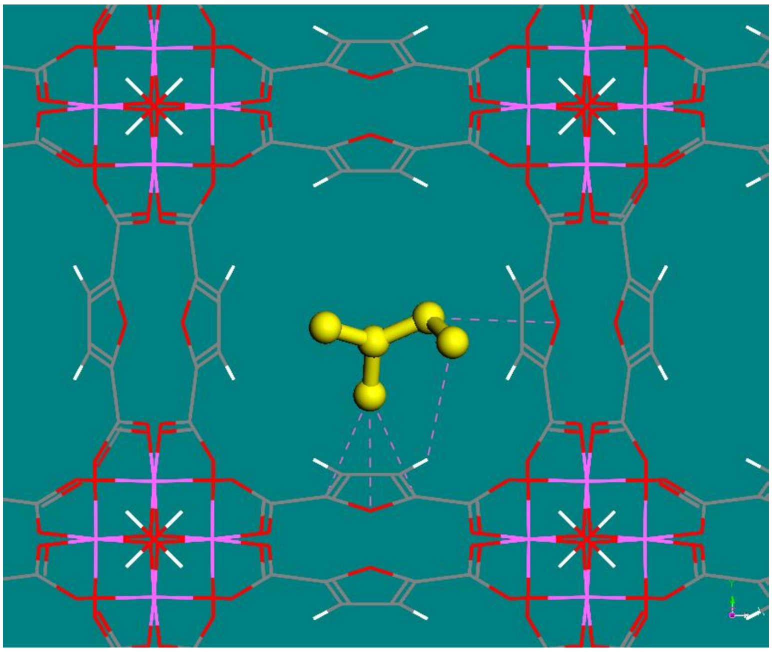

**Figure S40**. Illustration of a typical spatial arrangement and van der Waals close contacts (<3.5 Å) of an iC5 molecule with the MIL-160(Al) pore walls taken from a representative adsorption snapshot derived from CBMC simulation performed for an equimolar septenary mixture of all C5 and C6 isomers at *T* = 423 K and *P* = 20 kPa.

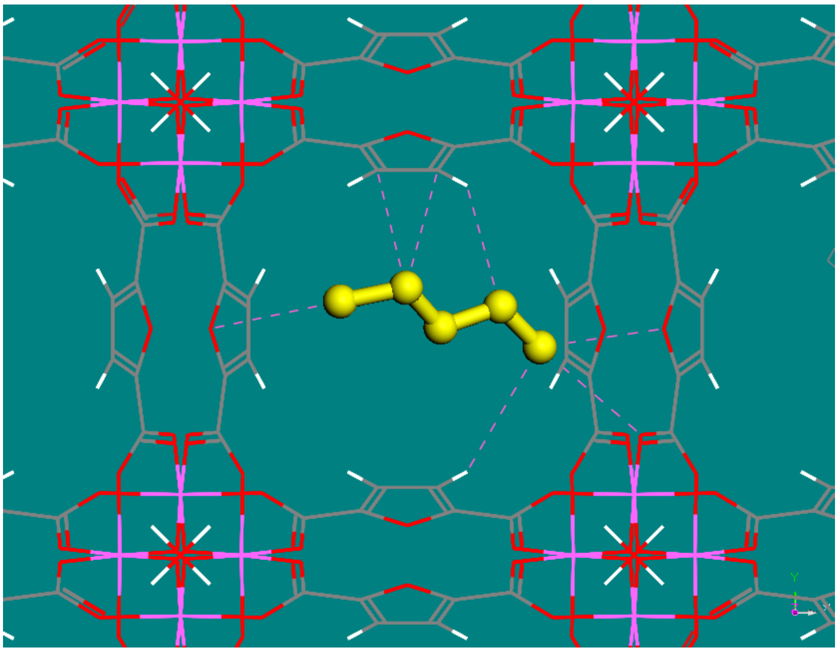

**Figure S41**. Illustration of a typical spatial arrangement and van der Waals close contacts (<3.5 Å) of an *n*-C5 molecule with the MIL-160(Al) pore walls taken from a representative adsorption snapshot derived from CBMC simulation performed for an equimolar septenary mixture of all C5 and C6 isomers at $T$ = 423 K and $P$ = 20 kPa.

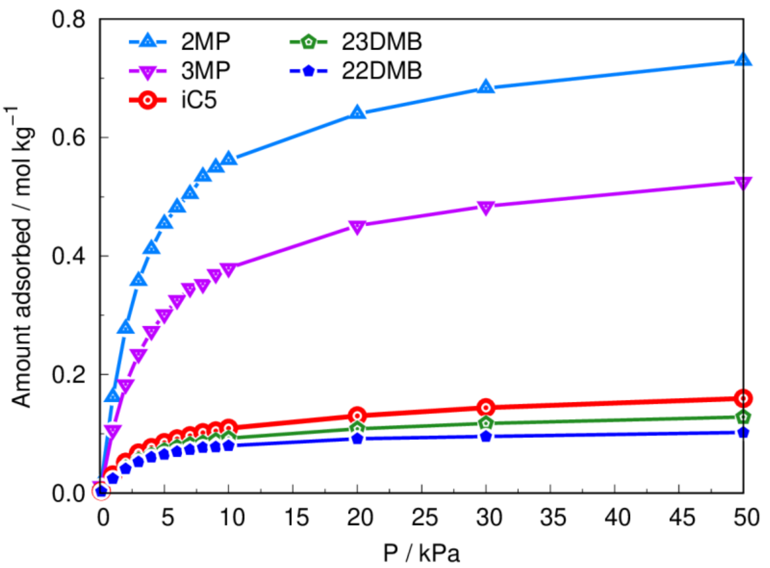


**Figure S42**. CBMC simulated co-adsorption isotherms of equimolar iC5/2MP/3MP/23DMB/22DMB mixture for the MIL-160(Al) with 5° tilted linkers at $T$ = 423 K.

**Table S1**. Textural properties of MIL-160(Al).

| Adsorbent | $S_{BET}$ ($m^2$ $g^{-1}$) | Median Pore (Å) | Maximum pore volume ($cm^3$ $g^{-1}$) |
|---|---|---|---|
| MIL-160(Al) | 1062 | 5.1 | 0.37 |

**Table S2**. Column, MFC´s, and syringe characteristics for the experimental studies.

| | |
|---|---|
| *Screening studies: powdered form* | |
| Column | 0.0046 m internal diameter and 0.10 m length |
| MFC | MC-5SCCM-D/5M |
| Syringe | 2.5 mL gastight syringe, 1002 TLL, PTFE Luer lock (Hamilton) |
| *Binary and ternary mixtures of C6 isomers: shaped form* | |
| Column | 0.01 m internal diameter and 0.12 m length |
| MFC | MC-100SCCM-D/5M |
| Syringe | 5.0 mL gastight syringe, 5MDF-LL-GT, PTFE Luer lock (SGE) |
| *Continuous cyclic experiments / quinary mixtures of C6 isomers: shaped form* | |
| Column | 0.01 m internal diameter and 0.42 m length |
| MFC | MC-100SCCM-D/5M |
| Syringe | 50 mL gastight syringe, 1050 TLL, PTFE Luer lock (Hamilton) |

**Table S3**. Molecular dimensions and research octane numbers of pentane and hexane isomers.

| C5/C6 Isomers | RON[5] | Molecular Shadow Length (Å)[12,13] | | | Isomerization effluent (mol %)[6] | Kinetic Diameter (Å)[14,15] |
|---|---|---|---|---|---|---|
| | | X | Y | Z | | |
| *n*-pentane (*n*-C5) | 61.7 | 9.1 | 4.5 | 4.0 | 25.9 | 4.3 |
| iso-pentane (iC5) | 93.5 | - | - | - | 32.0 | 5.0 |
| 2,2-dimethylbutane (22DMB) | 94 | 8.0 | 6.7 | 5.9 | 3.7 | 6.2 |
| 2,3-dimethylbutane (23DMB) | 105 | 7.8 | 6.7 | 5.3 | 2.9 | 5.6 |
| 2-methylpentane (2MP) | 74.5 | 9.2 | 6.4 | 5.3 | 12.8 | 5.0 |
| 3-methylpentane (3MP) | 75.5 | 9.3 | 6.2 | 5.2 | 8.5 | 5.0 |
| *n*-hexane (*n*-C6) | 30 | 9.7 | 4.5 | 4.0 | 14.1 | 4.3 |

**Table S4**. Experimental conditions to measure pure component breakthrough curves of hexane isomers on powder MIL-160(Al).

| Temperature (K) | Total isomers pressure (kPa) | Total isomers flowrate (μmol $min^{-1}$)[a)] | He flowrate (mL $min^{-1}$)[a)] | $q_{component}$ (mol $kg^{-1}$) | | | | |
|---|---|---|---|---|---|---|---|---|
| | | | | 22DMB | 23DMB | 3MP | 2MP | nC6 |
| *Single component: MIL-160(Al)* | | | | | | | | |
| 423 | 5.00 | 11.6 | 5.00 | 0.387 | 0.540 | 0.808 | 0.966 | 1.13 |
| 423 | 10.0 | 24.5 | 5.00 | 0.592 | 0.797 | 1.03 | 1.22 | 1.37 |
| 423 | 25.0 | 40.5 | 2.76 | 0.958 | 1.14 | 1.41 | 1.58 | 1.67 |
| 423 | 37.0 | 60.0 | 2.32 | 1.06 | 1.25 | 1.52 | 1.68 | 1.83 |
| 423 | 50.0 | 44.0 | 1.00 | 1.20 | 1.35 | 1.63 | 1.77 | 2.07 |
| | | | | | | | | |
| 473 | 5.00 | 11.6 | 5.00 | 0.0941 | 0.161 | 0.272 | 0.302 | 0.348 |
| 473 | 10.0 | 24.5 | 5.00 | 0.173 | 0.267 | 0.522 | 0.509 | 0.642 |
| 473 | 25.0 | 40.5 | 2.76 | 0.356 | 0.474 | 0.761 | 0.754 | 0.901 |
| 473 | 37.0 | 60.0 | 2.32 | 0.460 | 0.611 | 0.851 | 0.889 | 1.04 |
| 473 | 50.0 | 44.0 | 1.00 | 0.531 | 0.713 | 0.977 | 1.03 | 1.15 |

[a)] *The flowrates are given at the standard temperature and pressure conditions (STP);*

*The mass of MIL-160(Al) powder used in the experiments was 0.48*

**Table S5**. Experimental conditions to measure multicomponent breakthrough curves for an equimolar quinary mixture of hexane isomers on powder MIL-160(Al).

| Temperature (K) | Total isomers pressure (kPa) | Total isomers flowrate (μmol min$^{-1}$)[a)] | He flowrate (mL min$^{-1}$)[a)] | $q_{component}$ (mol kg$^{-1}$) | | | | | $q_{mixture}$ (mol kg$^{-1}$) | $s_{ads}$ |
|---|---|---|---|---|---|---|---|---|---|---|
| | | | | 22DMB | 23DMB | 3MP | 2MP | nC6 | | |
| *Quinary mixture: MIL-160(Al)* | | | | | | | | | | |
| 373 | 10.0 | 16.2 | 3.31 | 0.100 | 0.124 | 0.384 | 0.434 | 0.593 | 1.64 | 6.30 |
| 373 | 25.0 | 40.5 | 2.76 | 0.117 | 0.172 | 0.448 | 0.496 | 0.750 | 1.98 | 5.86 |
| 373 | 50.0 | 44.0 | 1.00 | 0.153 | 0.229 | 0.619 | 0.623 | 0.921 | 2.55 | 5.66 |
| 423 | 10.0 | 16.2 | 3.31 | 0.0760 | 0.118 | 0.256 | 0.291 | 0.395 | 1.14 | 4.86 |
| 423 | 25.0 | 14.7 | 1.00 | 0.0980 | 0.130 | 0.290 | 0.325 | 0.455 | 1.30 | 4.69 |
| 423 | 50.0 | 44.0 | 1.00 | 0.110 | 0.165 | 0.423 | 0.458 | 0.632 | 1.79 | 5.50 |
| 473 | 10.0 | 16.2 | 3.31 | 0.0289 | 0.0401 | 0.0905 | 0.0952 | 0.135 | 0.390 | 4.65 |
| 473 | 25.0 | 14.7 | 1.00 | 0.0545 | 0.0791 | 0.155 | 0.167 | 0.220 | 0.676 | 4.06 |
| 473 | 50.0 | 44.0 | 1.00 | 0.0642 | 0.104 | 0.214 | 0.216 | 0.319 | 0.917 | 4.45 |

*[a)] The flowrates are given at the standard temperature and pressure conditions (STP);*
*The mass of MIL-160(Al) powder used in the experiments was 0.48 g.*

**Table S6**. Experimental conditions to measure multicomponent breakthrough curves for an equimolar quinary mixture of hexane isomers on powder CAU-10(Al).

| Temperature (K) | Total isomers pressure (kPa) | Total isomers flowrate (μmol $min^{-1}$)[a)] | He flowrate (mL $min^{-1}$)[a)] | $q_{component}$ (mol $kg^{-1}$) | | | | | $q_{mixture}$ (mol $kg^{-1}$) |
|---|---|---|---|---|---|---|---|---|---|
| | | | | 22DMB | 23DMB | 3MP | 2MP | nC6 | |
| *Quinary mixture: CAU-10(Al)* | | | | | | | | | |
| 423 | 50.0 | 81.0 | 1.84 | 0.0802 | 0.115 | 0.254 | 0.209 | 0.198 | 0.856 |

[a)] *The flowrates are given at the standard temperature and pressure conditions (STP);*
*The mass of CAU-10 powder used in the experiments was 0.47 g.*

**Table S7**. Experimental conditions to measure multicomponent breakthrough curves for an equimolar quinary/septenary mixture of pentane and hexane isomers on powder MIL-160(Al).

| Temp. (K) | Total isomers pressure (kPa) | Total isomers flowrate (μmol min$^{-1}$)$^{a)}$ | He flowrate (mL min$^{-1}$)$^{a)}$ | $q_{component}$ (mol kg$^{-1}$) | | | | | | | $q_{mixture}$ (mol kg$^{-1}$) | $s_{ads}$ |
|---|---|---|---|---|---|---|---|---|---|---|---|---|
| | | | | iC5 | nC5 | 22DMB | 23DMB | 3MP | 2MP | nC6 | | |
| *Quinary mixture: MIL-160(Al)* | | | | | | | | | | | | |
| 373 | 50.0 | 81.0 | 1.84 | 0.165 | - | 0.172 | 0.251 | 0.697 | 0.650 | - | 1.94 | 2.29 |
| 423 | 50.0 | 44.0 | 1.00 | 0.147 | - | 0.125 | 0.201 | 0.453 | 0.491 | - | 1.42 | 2.00 |
| 473 | 50.0 | 44.0 | 1.00 | 0.0751 | - | 0.0685 | 0.107 | 0.213 | 0.230 | - | 0.694 | 1.77 |
| *Septenary mixture: MIL-160(Al)* | | | | | | | | | | | | |
| 373 | 50.0 | 81.0 | 1.84 | 0.117 | 0.196 | 0.0807 | 0.148 | 0.339 | 0.337 | 0.575 | 1.79 | 4.19 |
| 423 | 50.0 | 44.0 | 1.00 | 0.0943 | 0.184 | 0.0768 | 0.126 | 0.286 | 0.312 | 0.391 | 1.47 | 3.95 |
| 473 | 50.0 | 44.0 | 1.00 | 0.0752 | 0.123 | 0.0667 | 0.0973 | 0.165 | 0.177 | 0.239 | 0.943 | 2.94 |

[a)] *The flowrates are given at the standard temperature and pressure conditions (STP);*
*The mass of MIL-160(Al) powder used in the experiments was 0.48 g.*

**Table S8**. Experimental conditions to measure pure component breakthrough curves of pentane and hexane isomers on shaped MIL-160(Al).

| Temperature (K) | Total isomers pressure (kPa) | Total isomers flowrate (µmol min$^{-1}$)$^{a)}$ | He flowrate (mL min$^{-1}$)$^{a)}$ | $q_{component}$ (mol kg$^{-1}$) | | | | | | |
|---|---|---|---|---|---|---|---|---|---|---|
| | | | | iC5 | nC5 | 22DMB | 23DMB | 3MP | 2MP | nC6 |
| *Single component: MIL-160(Al)* | | | | | | | | | | |
| 373 | 2.50 | 12.2 | 10.8 | - | 0.873 | 0.677 | 0.733 | 0.999 | 1.11 | 1.24 |
| 373 | 5.00 | 24.3 | 10.5 | 0.784 | 1.09 | 0.753 | 0.909 | 1.15 | 1.28 | 1.32 |
| 373 | 10.0 | 48.6 | 9.93 | 1.01 | 1.25 | 0.939 | 1.17 | 1.33 | 1.40 | 1.47 |
| 373 | 20.0 | 97.2 | 8.83 | 1.29 | 1.42 | 1.10 | 1.34 | 1.55 | 1.60 | 1.66 |
| 373 | 30.0 | 146 | 7.73 | 1.48 | 1.54 | 1.18 | 1.45 | 1.64 | 1.67 | 1.70 |
| 373 | 50.0 | 243 | 5.21 | 1.58 | 1.63 | 1.29 | 1.49 | 1.67 | 1.75 | 1.80 |
| 423 | 2.50 | 10.7 | 9.49 | - | 0.292 | 0.170 | 0.229 | 0.366 | 0.471 | 0.579 |
| 423 | 5.00 | 21.4 | 9.25 | 0.263 | 0.440 | 0.263 | 0.403 | 0.519 | 0.656 | 0.721 |
| 423 | 10.0 | 42.9 | 8.76 | 0.431 | 0.653 | 0.405 | 0.529 | 0.716 | 0.779 | 0.899 |
| 423 | 20.0 | 85.7 | 7.79 | 0.599 | 0.821 | 0.575 | 0.712 | 0.908 | 0.990 | 1.10 |
| 423 | 30.0 | 129 | 6.81 | 0.733 | 0.929 | 0.675 | 0.819 | 0.983 | 1.07 | 1.19 |
| 423 | 50.0 | 214 | 4.87 | 0.922 | 1.09 | 0.805 | 0.955 | 1.14 | 1.22 | 1.28 |
| 473 | 2.50 | 9.58 | 8.49 | 0.0273 | 0.0508 | 0.0416 | 0.0610 | 0.0757 | 0.131 | 0.156 |
| 473 | 5.00 | 19.2 | 8.27 | 0.0609 | 0.143 | 0.0604 | 0.0967 | 0.125 | 0.201 | 0.255 |
| 473 | 10.0 | 38.3 | 7.83 | 0.130 | 0.223 | 0.130 | 0.167 | 0.238 | 0.290 | 0.394 |
| 473 | 20.0 | 76.7 | 6.96 | 0.214 | 0.344 | 0.205 | 0.313 | 0.424 | 0.498 | 0.561 |
| 473 | 30.0 | 115 | 6.09 | 0.321 | 0.442 | 0.273 | 0.390 | 0.579 | 0.603 | 0.705 |
| 473 | 50.0 | 192 | 4.35 | 0.435 | 0.529 | 0.363 | 0.482 | 0.685 | 0.728 | 0.822 |

*The flowrates are given at the standard temperature and pressure conditions (STP);*
*The mass of MIL-160(Al) shaped used in the experiments was 2.81 g.*

**Table S9**. Experimental conditions to measure multicomponent breakthrough curves for an equimolar binary/ternary mixture of hexane isomers on shaped MIL-160(Al).

| Temperature (K) | Total isomers pressure (kPa) | Total isomers flowrate (μmol $min^{-1}$)[a)] | He flowrate (mL $min^{-1}$)[a)] | $q_{component}$ (mol $kg^{-1}$) | | | | | $q_{mixture}$ (mol $kg^{-1}$) |
|---|---|---|---|---|---|---|---|---|---|
| | | | | 22DMB | 23DMB | 3MP | 2MP | nC6 | |
| *Binary mixture: MIL-160(Al)* | | | | | | | | | |
| 423 | 50.0 | 81.0 | 1.84 | 0.432 | 0.741 | - | - | - | 1.17 |
| 423 | 50.0 | 81.0 | 1.84 | - | 0.383 | - | 1.25 | - | 1.63 |
| 423 | 50.0 | 81.0 | 1.84 | - | - | 0.911 | 1.02 | - | 1.93 |
| 423 | 50.0 | 81.0 | 1.84 | - | - | - | 0.709 | 1.01 | 1.72 |
| *Ternary mixture: MIL-160(Al)* | | | | | | | | | |
| 423 | 50.0 | 81.0 | 1.84 | 0.239 | - | - | 0.723 | 0.971 | 1.93 |
| 423 | 50.0 | 81.0 | 1.84 | - | 0.183 | - | 0.600 | 0.846 | 1.63 |

[a)] *The flowrates are given at the standard temperature and pressure conditions (STP);*
*The mass of MIL-160(Al) shaped used in the experiments was 4.38 g.*

**Table S10**. Experimental conditions to measure multicomponent breakthrough curves for an equimolar quinary mixture of hexane isomers on shaped MIL-160(Al).

| Temperature (K) | Total isomers pressure (kPa) | Total isomers flowrate (μmol min$^{-1}$)[a)] | He flowrate (mL min$^{-1}$)[a)] | $q_{component}$ (mol kg$^{-1}$) | | | | | $q_{mixture}$ (mol kg$^{-1}$) | $s_{ads}$ |
|---|---|---|---|---|---|---|---|---|---|---|
| | | | | 22DMB | 23DMB | 3MP | 2MP | nC6 | | |
| *Quinary mixture: MIL-160(Al)* | | | | | | | | | | |
| 423 | 50.0 | 281 | 6.39 | 0.0572 | 0.116 | 0.328 | 0.378 | 0.542 | 1.42 | 7.20 |
| 473 | 50.0 | 252 | 5.71 | 0.0445 | 0.0868 | 0.183 | 0.203 | 0.278 | 0.795 | 5.06 |

[a)] *The flowrates are given at the standard temperature and pressure conditions (STP);*
*The mass of MIL-160(Al) shaped used in the experiments was 16.5 g.*

**Table S11**. Experimental conditions to measure multicomponent breakthrough curves for a septenary mixture of pentane and hexane isomers on shaped MIL-160(Al) with an isomerization concentration.[6]

| Temperature (K) | Total isomers pressure (kPa) | Total isomers flowrate (μmol min$^{-1}$)$^{a)}$ | He flowrate (mL min$^{-1}$)$^{a)}$ | $q_{component}$ (mol kg$^{-1}$) | | | | | | | $q_{mixture}$ (mol.kg$^{-1}$) |
|---|---|---|---|---|---|---|---|---|---|---|---|
| | | | | iC5 | nC5 | 22DMB | 23DMB | 3MP | 2MP | nC6 | |
| *Quinary mixture: MIL-160(Al)* | | | | | | | | | | | |
| 423 | 50.0 | 81.0 | 1.84 | 0.257 | - | 0.0184 | 0.0263 | 0.214 | 0.355 | - | 0.871 |
| *Septenary mixture: MIL-160(Al)* | | | | | | | | | | | |
| 423 | 50.0 | 81.0 | 1.84 | 0.142 | 0.228 | 0.0133 | 0.0144 | 0.110 | 0.187 | 0.284 | 0.979 |

[a)] *The flowrates are given at the standard temperature and pressure conditions (STP);*
*The mass of MIL-160(Al) shaped used in experiments was 5.27 g.*

**Table S12**. Experimental conditions to measure multicomponent breakthrough curves for an equimolar septenary mixture of pentane and hexane isomers in a mixed bed of shaped MOF MIL-160(Al) (70 wt%) and binder-free beads Zeolite 5A (30 wt%).

| Temp. (K) | Total isomers pressure (kPa) | Total isomers flowrate (μmol $min^{-1}$)[a)] | He flowrate (mL $min^{-1}$)[a)] | $q_{component}$ (mol $kg^{-1}$) | | | | | | | $q_{mixture}$ (mol $kg^{-1}$) | $s_{ads}$ |
|---|---|---|---|---|---|---|---|---|---|---|---|---|
| | | | | iC5 | nC5 | 22DMB | 23DMB | 3MP | 2MP | nC6 | | |
| *Septenary mixture: MIL-160(Al) + Zeolite 5A* | | | | | | | | | | | | |
| 423 | 50.0 | 563 | 12.8 | 0.0821 | 0.247 | 0.0805 | 0.135 | 0.317 | 0.356 | 0.623 | 1.84 | 5.18 |
| 473 | 50.0 | 503 | 11.4 | 0.0400 | 0.160 | 0.0439 | 0.0764 | 0.163 | 0.173 | 0.388 | 1.04 | 5.51 |

*[a)] The flowrates are given at the standard temperature and pressure conditions (STP);*
*The mass of MIL-160(Al) and Zeolite 5A shaped used in the mixed-bed experiments was 12.8 g and 5.49 g, respectively.*

**Table S13**. Experimental conditions for preliminary PSA tests for an equimolar septenary mixture of pentane and hexane isomers in a mixed bed of shaped MOF MIL-160(Al) (70 wt%) and binder-free beads Zeolite 5A (30 wt%).

| Temperature (K) | Total isomers pressure (kPa) | Total isomers flowrate (μmol $min^{-1}$)[a)] | He flowrate (feed) (mL $min^{-1}$)[a)] | He flowrate (purge) (mL $min^{-1}$)[a)] | Number of Cycles | Adsorption step time (min) | Desorption step time (min) | Total Duration (min) |
|---|---|---|---|---|---|---|---|---|
| 423 | 50.0 | 563 | 12.8 | 76.7 | 6 | 33.0 | 18.0 | 306 |
| 473 | 50.0 | 503 | 11.4 | 68.6 | 10 | 24.0 | 12.0 | 360 |

[a)] *The flowrates are given at the standard temperature and pressure conditions (STP);*

*The mass of MIL-160(Al) and Zeolite 5A shaped used in the mixed-bed experiments was 12.8 g and 5.49 g, respectively.*

**Table S14**. Intermolecular LJ-potential parameters for the pentane and hexane isomers.

| Pseudo atom type | σ (Å) | ε/$k_B$ (K) |
|---|---|---|
| $CH_3$ | 3.75 | 98.0 |
| $CH_2$ | 3.95 | 46.0 |
| CH | 4.68 | 10.0 |
| C | 6.40 | 0.5 |

**Table S15**. Intermolecular LJ Potential parameters for the MIL-160(Al) framework atoms.

| Atom type | $\sigma$ (Å) | $\varepsilon/k_B$ (K) |
|---|---|---|
| H | 2.571 | 0 |
| C | 52.839 | 4.008 |
| O | 30.194 | 3.118 |
| Al | 4.008 | 0 |

**Table S16**. DFT-calculated host-guest binding energies and CBMC calculated Henry constants ($K_H$) for the adsorbed C5-C6 alkane isomers in the MIL-160(Al).

| Alkane | B.E. (kJ $mol^{-1}$) | $K_H$ (mol $kg^{-1}$ $Pa^{-1}$) |
|---|---|---|
| *n*-C6 | -85.78 | 1.18 x $10^{-3}$ |
| 2MP | -82.33 | 8.26 x $10^{-4}$ |
| 3MP | -81.58 | 6.11 x $10^{-4}$ |
| *n*-C5 | -75.22 | 3.80 x $10^{-4}$ |
| 23DMB | -72.97 | 1.36 x $10^{-4}$ |
| *i*-C5 | -69.95 | 1.74 x $10^{-4}$ |
| 22DMB | -61.14 | 1.34 x $10^{-4}$ |

## REFERENCES


[1] A. Cadiau, J. S. Lee, D. Damasceno Borges, P. Fabry, T. Devic, M. T. Wharmby, C. Martineau, D. Foucher, F. Taulelle, C. H. Jun, Y. K. Hwang, N. Stock, M. F. De Lange, F. Kapteijn, J. Gascon, G. Maurin, J. S. Chang, C. Serre, *Adv. Mater.* **2015**, *27*, 4775.

[2] M. Thommes, K. Kaneko, A. V. Neimark, J. P. Olivier, F. Rodriguez-Reinoso, J. Rouquerol, K. S. W. Sing, *Pure Appl. Chem.* **2015**, *87*, 1051.

[3] J. S. Chang, U. H. Lee, Y. K. Hwang, C. Serre, T. Devic, A. Cadiau, N. Stock, N. Reimer, M. T. Wharmby, *Adsorbents Comprising Organic-Inorganic Hybrid Nanoporous Materials for Sorption of Water or Alcohol and Use Thereof*, **2016**.

[4] A. Henrique, A. E. Rodrigues, J. A. C. Silva, *Ind. Eng. Chem. Res.* **2019**, *58*, 378.

[5] Z. R. Herm, B. M. Wiers, J. A. Mason, J. M. Van Baten, M. R. Hudson, P. Zajdel, C. M. Brown, N. Masciocchi, R. Krishna, J. R. Long, *Science (80-. ).* **2013**, *340*, 960.

[6] T. C. Holcombe, T. C. Sager, W. K. Volles, A. S. Zarchy, *Isomerization Process. U.S. Patent 4,929,799*, **1990**, 4,929,799.

[7] A. Henrique, T. Maity, H. Zhao, P. F. Brântuas, A. E. Rodrigues, F. Nouar, A. Ghoufi, G. Maurin, J. A. C. Silva, C. Serre, *J. Mater. Chem. A* **2020**, *8*, 17780.

[8] D. Dubbeldam, S. Calero, D. E. Ellis, R. Q. Snurr, *Mol. Simul.* **2016**, *42*, 81.

[9] M. G. Martin, J. I. Siepmann, *J. Phys. Chem. B* **1998**, *102*, 2569.

[10] M. G. Martin, J. I. Siepmann, *J. Phys. Chem. B* **1999**, *103*, 4508.

[11] A. K. Rappé, C. J. Casewit, K. S. Colwell, W. A. Goddard, W. M. Skiff, *J. Am. Chem. Soc.* **1992**, *114*, 10024.

[12] D. Peralta, G. Chaplais, A. Simon-Masseron, K. Barthelet, G. D. Pirngruber, *Ind. Eng. Chem. Res.* **2012**, *51*, 4692.

[13] C. E. Webster, R. S. Drago, M. C. Zerner, *J. Am. Chem. Soc.* **1998**, *120*, 5509.

[14] M. L. Maloncy, L. Gora, J. C. Jansen, T. Maschmeyer, *Ars Sep. Acta* **2003**, *2*, 18.

[15] Ş. B. Tantekin-Ersolmaz, L. Şenorkyan, N. Kalaonra, M. Tatlier, A. Erdem-Şenatalar, *J. Memb. Sci.* **2001**, *189*, 59.
